# Multimodal Temporal Pattern Discrimination Is Encoded in Visual Cortical Dynamics

**DOI:** 10.1523/ENEURO.0047-23.2023

**Published:** 2023-07-24

**Authors:** Sam Post, William Mol, Omar Abu-Wishah, Shazia Ali, Noorhan Rahmatullah, Anubhuti Goel

**Affiliations:** Department of Psychology, University of California, Riverside, Riverside, California 92521

**Keywords:** two-photon, audiovisual temporal patterns, temporal discrimination, temporal learning, visual cortical dynamics

## Abstract

Discriminating between temporal features in sensory stimuli is critical to complex behavior and decision-making. However, how sensory cortical circuit mechanisms contribute to discrimination between subsecond temporal components in sensory events is unclear. To elucidate the mechanistic underpinnings of timing in primary visual cortex (V1), we recorded from V1 using two-photon calcium imaging in awake-behaving mice performing a go/no-go discrimination timing task, which was composed of patterns of subsecond audiovisual stimuli. In both conditions, activity during the early stimulus period was temporally coordinated with the preferred stimulus. However, while network activity increased in the preferred condition, network activity was increasingly suppressed in the nonpreferred condition over the stimulus period. Multiple levels of analyses suggest that discrimination between subsecond intervals that are contained in rhythmic patterns can be accomplished by local neural dynamics in V1.

## Significance Statement

Judging whether to stop or go through a yellow light requires determining the duration of the yellow light, and language users must produce sequences of syllables in a temporally structured manner: thus, the ability to tell time is critical. An emerging hypothesis is that local changes in neural activity can contain information about time in the subsecond range. Based on prior human experiments, we have designed a novel timing task for mice and show that mice learn to discriminate between two temporal patterns of audiovisual stimuli. Task performance is accompanied by visual cortical circuit mechanisms. By combining cutting-edge tools with simple behavior, we provide fundamental insight into the neural mechanisms of timing which will also guide future therapies for timing deficits.

## Introduction

A key aspect of sensory discrimination in learning and memory and in generating complex behavior is extracting temporal features from external stimuli. For example, one may need to keep a beat and synchronize tempo when in a band; a prey may need to jump out of the way of a predator at just the right moment; the timing of a yellow light must be predicted to decide whether to slow down or to go through it; and meaning in spoken language derives from sequences of syllables that are highly temporally structured. Based on psychophysical and pharmacological data, it is most likely that there are multiple neural mechanisms that code for the temporal structure of sensory events since they are timed over a broad range of scales, ranging from microseconds to days (Mauk and Buonomano, 2004; Buhusi and Meck, 2005; Paton and Buonomano, 2018). However, a growing body of literature suggests that time intervals in the subsecond and second range are encoded in the emergent changing patterns or neural dynamics across many brain areas, including sensory cortex (Pastalkova et al., 2008; Carnevale et al., 2015; Gouvêa et al., 2015; Namboodiri et al., 2015; Goel and Buonomano, 2016; Soares et al., 2016; Bakhurin et al., 2017; Emmons et al., 2017; Heys and Dombeck, 2018; Tsao et al., 2018; Zhou et al., 2020; Tonoyan et al., 2022).

In the traditional hierarchical view of brain organization, the role of primary sensory cortex is to generate a reliable representation of the sensory world, and sensory representations are then decoded by higher-order areas (Hubel and Wiesel, 1962; Felleman and Van Essen, 1991; Miller and Cohen, 2001). Other studies suggest a more active involvement that shapes sensory perception (Glickfeld et al., 2013; Znamenskiy and Zador, 2013); a large body of experimental evidence has now shown that sensory areas contribute to several “higher-order” nonsensory features (Gordon and Stryker, 1996; Shuler and Bear, 2006; Niell and Stryker, 2010; Zhou et al., 2010; Brosch et al., 2011; Zelano et al., 2011; Keller et al., 2012; Gavornik and Bear, 2014a, b; Namboodiri et al., 2015), such as timing and temporal context. Although the locus of temporal predictions and subsecond and second timing has traditionally been attributed to higher-order cortical areas (Leon and Shadlen, 2003; Jazayeri and Shadlen, 2015; Licata et al., 2017) and subcortical areas (Bakhurin et al., 2017; Zhou et al., 2020; Toso et al., 2021), accumulating evidence suggests that primary visual cortex (V1) exhibits response modulation to “higher” functions such as spatiotemporal learning as well as reward prediction and attention (Gavornik and Bear, 2014a). Specifically, Shuler and Bear (2006) showed that as rats perform a visually cued timing task, V1 cortical activity rapidly modulated to predict the arrival of reward. Additionally, cholinergic function contributed to the modification of V1 activity (Chubykin et al., 2013). Namboodiri et al. (2015) used a similar task and showed that indeed cortical activity in V1 reflected the duration of a target interval.

While most studies implement timing tasks as discrete durations or time intervals, temporal structure in sensory stimuli is often organized as sequential events. Sequences may be composed of simple isochronous stimuli and intervals between stimuli, as in rhythms, or complex arrangements of varying stimulus and interval durations, such as in language and music. However, the neural dynamic regimes in the sensory cortex that contribute to processing and learning rhythmic patterns remain largely unclear. One prevailing idea is that neural oscillations allow communication between sensory and motor cortical areas, thus producing temporal predictions and entrainment to rhythms (Merchant et al., 2015). Specifically, do emergent neural dynamics in V1 contribute to learning the temporal structure of rhythmic patterns? To understand how visual cortical dynamics adapt to the temporal structure of a multimodal rhythm in a goal-directed task, we implemented a novel audiovisual (AV) timing task, temporal pattern sensory discrimination (TPSD), in awake behaving mice using two-photon calcium imaging in V1, layer 2/3 (L-2/3). In the TPSD task, mice learn to discriminate between two temporal patterns. Our paradigm builds on previous work in temporal pattern discrimination, which suggests that multisensory stimuli enhance the discriminability of sequences (Raposo et al., 2012; Barakat et al., 2015). Examination of visual cortex as a locus of change, in an audiovisual task, was influenced by studies showing modulation of visual cortical plasticity by functional input from other brain areas such as hippocampus (Finnie et al., 2021) and auditory cortex (McIntosh et al., 1998; Zangenehpour and Zatorre, 2010; Deneux et al., 2019; Garner and Keller, 2022). Studies have also shown that audiovisual stimuli evoke multimodal plasticity in V1 (Morrell, 1972; Petro et al., 2017).

Here, we show that mice can discriminate between two temporal patterns to achieve expert status on a goal-directed task and that learning was accompanied by robust changes in visual cortical dynamics that reflected the temporal structure of the experienced rhythms. Further, using multiple analyses we show that emergent activity in V1 contributes to trial outcomes. In conclusion, this study underscores the hypothesis that intrinsic network mechanisms contribute to learning and representation of temporal patterns.

## Materials and Methods

### Experimental animals

All experiments followed the US National Institutes of Health GUIDE for the Care and Use of Laboratory Animals, under animal use protocols approved by the Chancellor’s Animal Research Committee and Office for Animal Research Oversight at the University of California, Riverside (ARC #2022–0022). We used male and female FVB.129P2 (FVB) WT mice (stock #004828, The Jackson Laboratory). All mice were housed in a vivarium with a 12 h light/dark cycle, and experiments were performed during the light cycle. The FVB background was chosen because of its robust breeding.

### Go/no-go TPSD task for head restrained mice

**Figure 1.**
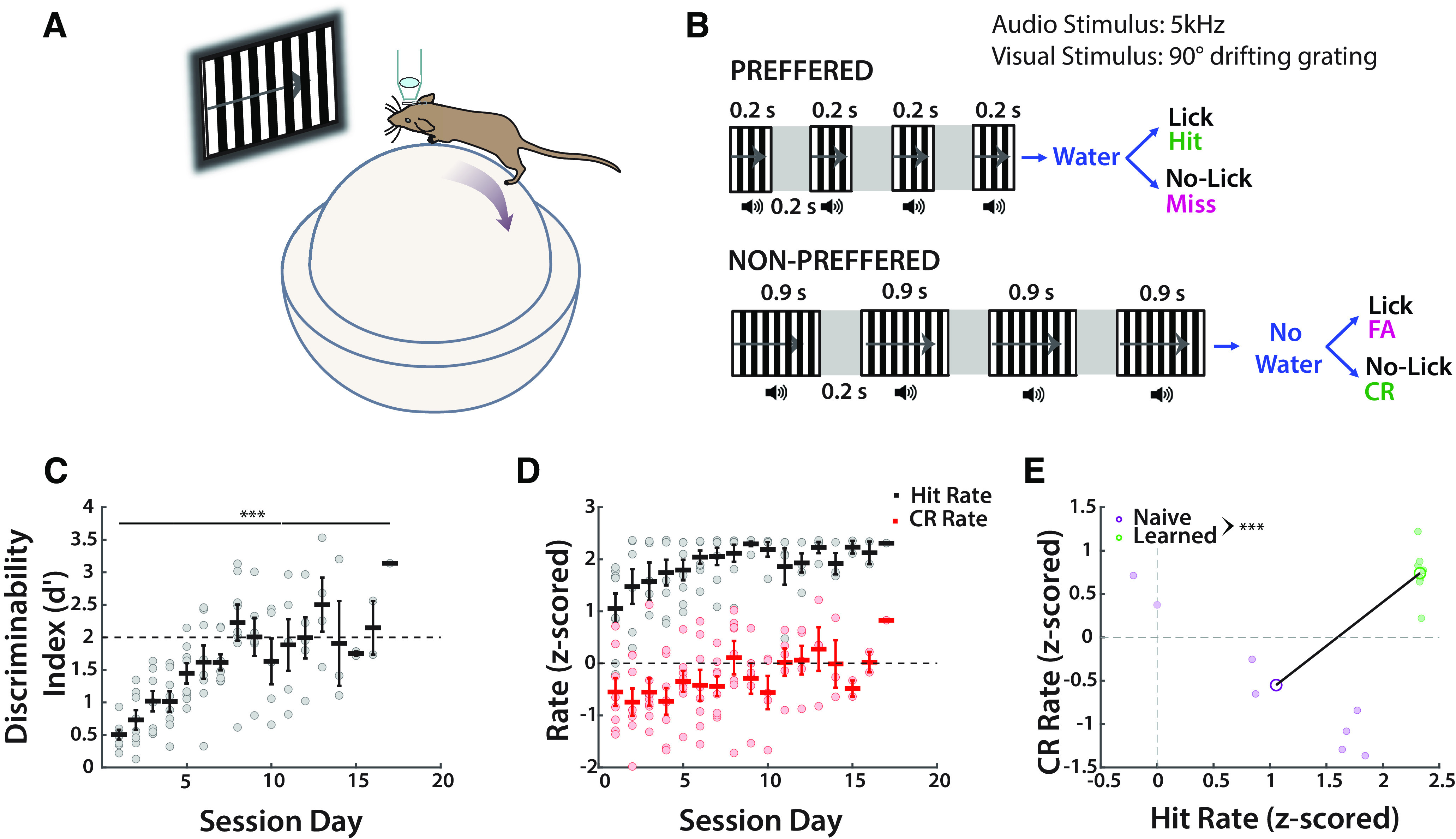
Mice achieve expert status on the TPSD task (*n* = 8). ***A***, Schematic of mouse on polystyrene ball. ***B***, Experimental paradigm is a go/no-go task composed of synchronous audiovisual stimuli. ***C***, *d′* shows mice learn to discriminate temporal patterns (one-way ANOVA: *F*_(1,16)_ = 5.45, *p* = 1.23 × 10^−7^). ***D***, Hrs and CRrs do not significantly change with learning (Hr: Kruskal–Wallis test: *H*_(16)_ = 21.74. *p* = 0.1515; CRr one-way ANOVA: *F*_(1,16)_ = 1.23, *p* = 0.26). ***E***, Hr and CRr in naive and learned sessions are significantly different (Hr: two-tailed *t* test: *t*_(14)_ = 4.42, *p* = 5.85 × 10^−4^; CRr: two-tailed *t* test: *t*_(14)_ = 4.46, *p* = 5.3 × 10^−4^). Refer to Extended Data Figures 1-1, 1-2, 1-3, 1-4, which show no dependence on trial ratios or training paradigm. The Extended Data also show dependence on the stimulus for learning.

Awake, head-restrained young adult mice (2–4 months) were allowed to run on an air-suspended polystyrene ball while performing the task in our custom-built rig (Fig. 1*A*). Before performing the task, the animals were subjected to handling, habituation, and pretrial phases. After recovery from headbar/cranial window surgery, mice were handled gently for 5 min every day, until they were comfortable with the experimenter and would willingly transfer from one hand to the other to eat sunflower seeds. This was followed by water deprivation (giving mice a rationed supply of water once per day) and habituation to the behavior rig. During habituation, mice were head restrained and acclimated to the enclosed soundproof chamber and allowed to run freely on the 8 cm polystyrene ball. Eventually, mice were introduced to the lickport that dispensed water (3–4 μL) and recorded licking [custom-built at the University of California, Los Angeles (UCLA) electronics shop], followed by the audiovisual stimuli. This was repeated for 10 min per session for 3 d. Starting water deprivation before pretrials motivated the mice to lick (Guo et al., 2014). After habituation and an ∼15% weight loss, mice started the pretrial phase of the training. During pretrials, mice were shown the preferred stimulus only with no punishment time associated with incorrect responses. This was done (1) to teach the mice the task structure and (2) to encourage the mice to lick and to remain motivated. The first day consisted of 150 trials, and subsequent days of 250 trials. The reward, as in the TPSD main task, was dispensed at 1.2 s and remained available to the mice until 2 s, at which time it was sucked away by a vacuum. The mice were required to learn to associate a water reward soon after the stimulus was presented and that there was no water reward in the intertrial interval (4 s period between trials). Initially during pretrials, the experimenter pipetted small drops of water onto to the lickport to coax the mice to lick. Once the mice learned this and licked with 80% efficiency, they were advanced to the go/no-go task.

10.1523/ENEURO.0047-23.2023.f1-1 Figure 1-1 Learning is sustained regardless of the preferred to nonpreferred trial ratio (*n* = 8). ***A***, Discriminability index (two-tailed *t* test: *t*_(14)_ = 1.4175, *p* = 0.1782), Hit rate (two-tailed *t* test: *t*_(14)_ = 2.099, *p* = 0.0544), and CR rate (two-sided Wilcoxon rank-sum test: *p* = 0.6454) do not significantly differ between learned sessions in main trials (P/NP stimulus ratio, 7:3) and 6:4 P/NP stimulus ratio sessions, indicating nonbiased learning. ***B***, Rasters of licking in learned main sessions and 6:4 P/NP stimulus ratio sessions. ***C***, Probability of a licking event as a function of time by stimulus type and session. ***D***, Accuracy of bootstrapped SVM as a function of time. Licking events per 0.067 s were the predictors, and stimulus type was the outcome. Comparable predictability between learned and P/NP stimulus ratio 6:4 sessions indicate nonbiased learning. Download Figure 1-1, EPS file.

**Figure 2.**
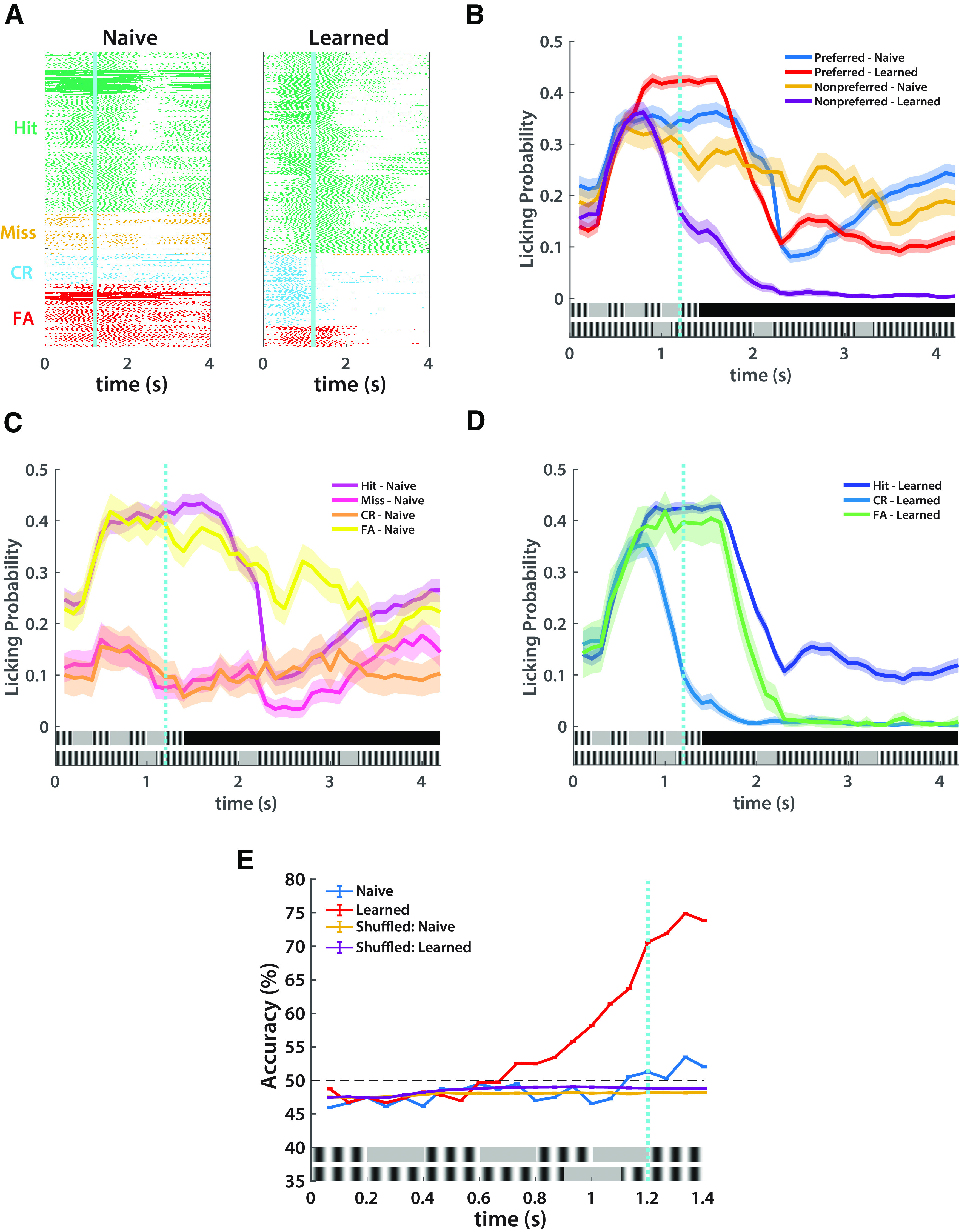
Licking profiles index learning (*n* = 8). ***A***, Raster plots of licking in naive and learned sessions, using the best 150 trials of all mice, as determined by the best *d′* for that session. ***B***, Probability of a lick event by stimulus type and session. ***C***, Probability of a lick event by trial outcome in naive sessions. ***D***, Probability of a lick event by trial outcome in learned sessions. Miss trials are excluded as there were exceedingly few miss trials for each mouse. ***E***, Accuracy of bootstrapped SVM as a function of time. Licking events per 0.067 s were the predictors, and stimulus type is the outcome. Learned session accuracy confirms learning as predictability rises above chance before the water reward at 1.2 s.

10.1523/ENEURO.0047-23.2023.f1-2 Figure 1-2 Learning is stimulus dependent (*n* = 7). ***A***, Discriminability index (two-tailed *t* test: *t*_(13)_ = 11.0036, *p* = 5.86 × 10^−8^), Hit rate (two-tailed *t* test: *t*_(13)_ = 24.2266, *p* = 3.34 × 10^−12^), and CR rate (two-tailed *t* test: *t*_(13)_ = 4.0389, *p* = 0.0014) significantly differ between learned sessions in main trials and control sessions in which both monitor and speaker are turned off, indicating that learning is stimulus dependent. ***B***, Rasters of licking in learned main sessions and control sessions. ***C***, Probability of licking event as a function of time by stimulus type and session. ***D***, Accuracy of bootstrapped SVM as a function of time. Licking events per 0.067 s were the predictors, and stimulus type is the outcome. Predictability at chance level in control sessions indicates that learning is stimulus dependent. Download Figure 1-2, EPS file.

**Figure 3.**
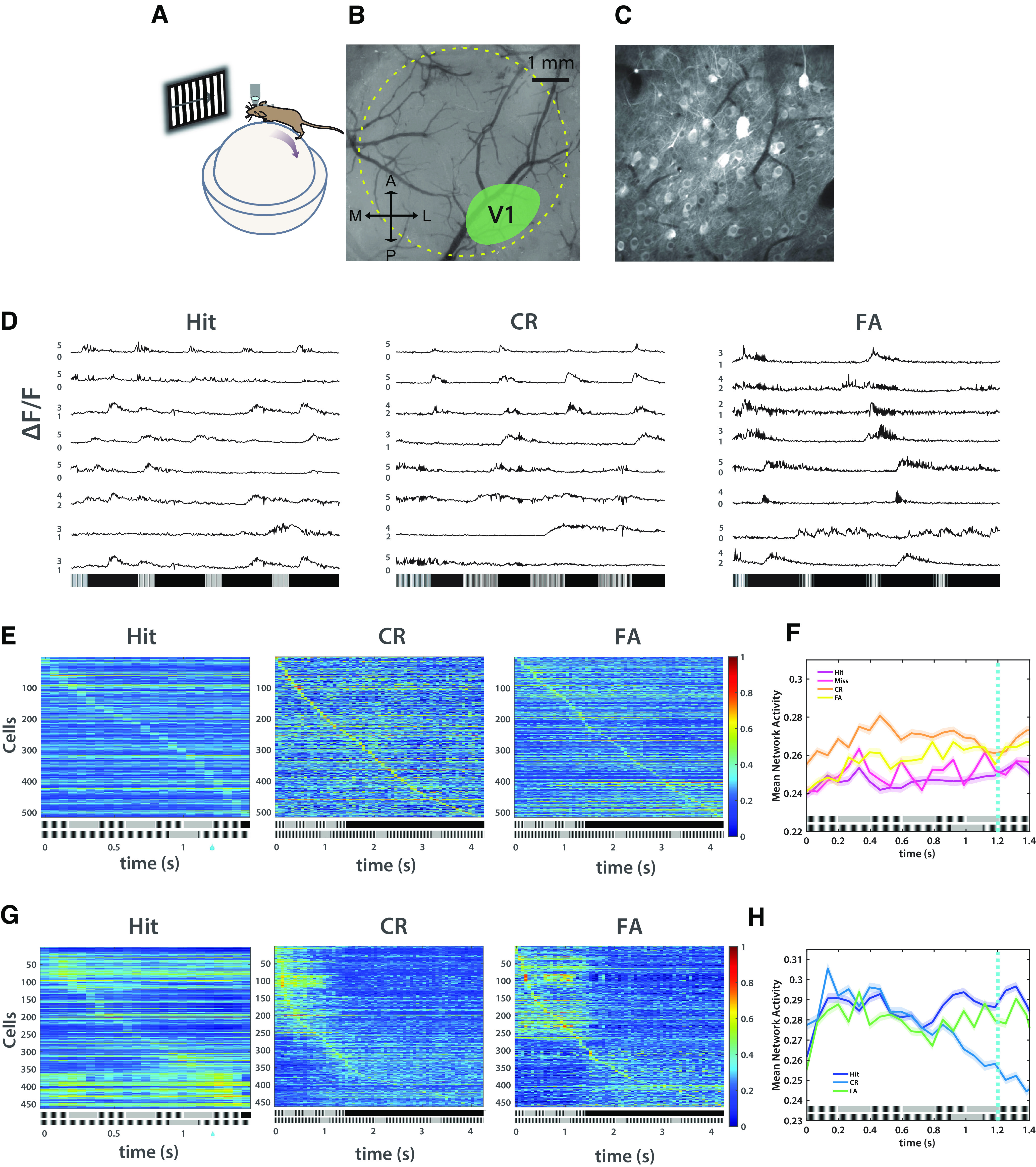
V1 neural activity changes with learning and represents stimuli and trial outcomes (*n* = 5). ***A***, Schematic of a mouse on a polystyrene ball with microscope objective over V1. ***B***, Example craniotomy window showing binocular V1. ***C***, Example frame of video taken from binocular V1 during behavior with imaging. ***D***, Raw florescence traces of 10 representative cells in one mouse over 3 trial outcomes in learned session. ***E***, Spike-sorted mean activity of all non-lick-modulated cells, in all trials, in naive sessions. Shaded areas represent 95% confidence intervals. ***F***, Mean spiking activity in naive sessions. ***G***, Spike-sorted mean activity of all non-lick-modulated cells, in all trials, in learned sessions. ***H***, Mean spiking activity in learned sessions. Shaded areas represent 95% confidence intervals. Refer to Extended Data Figures 3-1 and 3-2 for extended analysis on neural activity from V1 during learning. Extended Data Figures 3-3 and 3-4 show the analysis of lick-modulated cells. Refer to Extended Data Figure 3-5 for learned neural dynamics during the flipped paradigm.

10.1523/ENEURO.0047-23.2023.f1-3 Figure 1-3 Mice achieve expert status on the TPSD_mod_ paradigm. ***A***, Schematic of flipped paradigm. Synchronous audiovisual stimuli are presented as before in the original paradigm. The preferred stimulus has the longer intratrial stimulus of 0.73 s; the nonpreferred is composed of 0.2 s intratrial stimuli. The total time between stimuli is now equal at 2.6 s. ***B***, Raster plot of licking between naive and learned sessions (*n* = 2). ***C***, Discriminability index across days shows learning in mice. ***D***, CR and Hit rates change with sessions. ***E***, Change in performance is driven primarily by changes in CR rates. ***F***, Probabilities of licking by stimulus type and session day. ***G***, Probabilities of licking in naive sessions. ***H***, Probabilities of licking in learned sessions. Miss trials are removed as there were exceedingly few. ***I***, SVM accurately predicts stimuli from licking data as a function of time in learned sessions. Naive predictability remains at chance level until after the period at which the water reward is delivered. Download Figure 1-3, EPS file.

**Figure 4.**
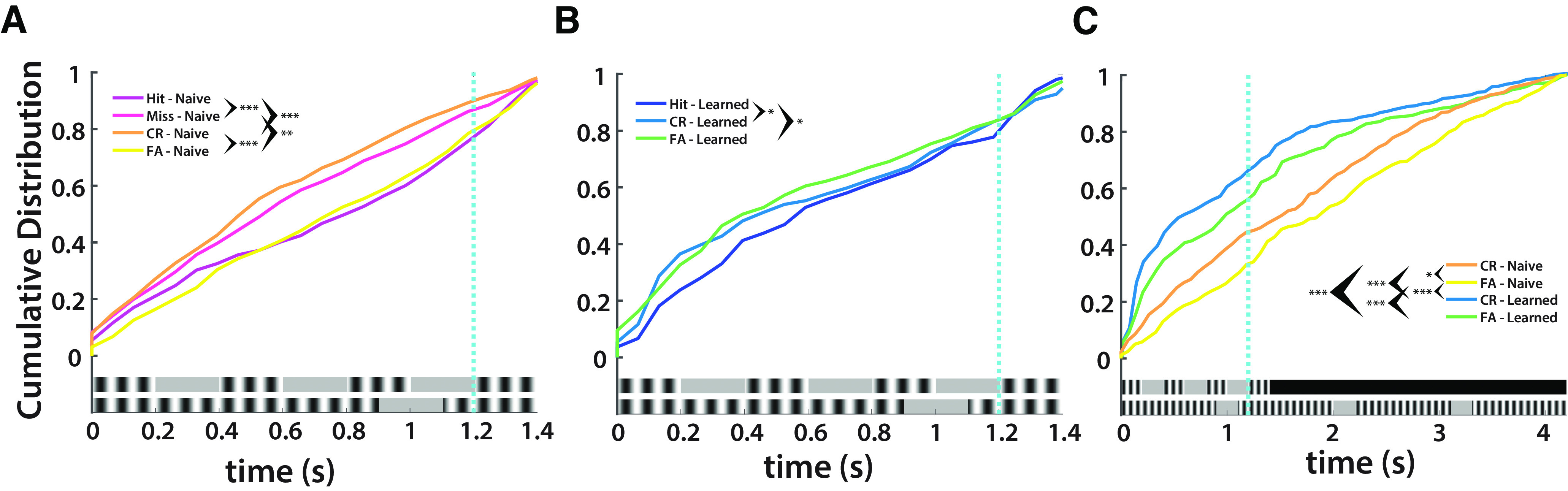
V1 neural activity ramps earlier in learned sessions (*n* = 5). ***A***, Cumulative Distribution Function (CDF) of maximum spiking in naive sessions by trial outcome in preferred stimulus period (KS tests with Bonferroni correction: α = 0.0083; Hit vs Miss: *D*(0.1609), *p* = 2.58 × 10^−6^; Hit vs CR: *D*(0.2035), *p* = 7.49 × 10^−10^; Hit vs FA: *D*(0.062), *p* = 0. 2658; Miss vs CR: *D*(0.064), *p* = 0.234; Miss vs FA: *D*(0.1434), *p* = 4.16 × 10^−5^; CR vs FA: *D*(0.1899), *p* =1.23 × 10^−8^). ***B***, CDF of maximum spiking in learned sessions by trial outcome in preferred stimulus period (KS tests with Bonferroni correction: α = 0.0167; Hit vs CR: *D*(0.1274), *p* = 9.57 × 10^−4^; Hit vs FA: *D*(0.1339), *p* = 4.31 × 10^−4^; CR vs FA: *D*(0.0518), *p* = 0.5518). ***C***, CDF of maximum spiking in naive and learned sessions by trial outcome in nonpreferred stimulus period (KS tests with Bonferroni correction: α = 0.0083; Naive CR vs Naive FA: *D*(0.124), *p* = 6.29 × 10^−4^; Naive CR vs Learned CR: *D*(0.2612), *p* = 4.01 × 10^−15^; Naive CR vs Learned FA: *D*(0.1873), *p* = 5.57 × 10^−8^; Naive FA vs Learned CR: *D*(0.3369), *p* = 7.16 × 10^−25^; Naive FA vs Learned FA: *D*(0.2478), *p* = 1.19 × 10^−13^; Learned CR vs Learned FA: *D*(0.1058), *p* = 0.01).

10.1523/ENEURO.0047-23.2023.f1-4 Figure 1-4 Learning on the TPSD_mod_ paradigm is not an artifact of experimental design. ***A***, Discriminability index, Hit rates, and CR rates in learned and P/NP stimulus ratio 6:4 sessions (*n* = 2). ***B***, Raster plot of licking between learned and P/NP stimulus ratio 6:4 sessions. ***C***, Probabilities of licking by stimulus type and session day. ***D***, SVM accuracy in P/NP stimulus ratio 6:4 session mirrors SVM accuracy using licking to predict stimulus type in learned sessions, confirming that the main task P/NP stimulus ratio 7:3 is not a confound. ***E***, Discriminability index, Hit rates, and CR rates in learned and control (monitor and speakers turned off) sessions (*n* = 2). ***B***, Raster plot of licking between learned and control sessions. ***C***, Probabilities of licking by stimulus type and session day. ***D***, SVM accuracy using licking to predict stimulus type in control sessions remains at chance level throughout the trial period confirming that learning is stimulus dependent. Download Figure 1-4, EPS file.

Control sessions confirmed that learning was stimulus dependent: expert performance on the task required the audiovisual stimuli and was not simply dependent on the availability of a water reward. *d′*, CRr, and Hr were all significantly different from the main task, showing poor performance (Extended Data Fig. 1-2*A*). Licking profiles showed considerable changes, both in the volume of licking and in stimulus-dependent licking (Extended Data Fig. 1-2*B*,*C*). Using licking in Control trials to predict stimuli via the bootstrapped SVM showed chance performance throughout the trial period (Extended Data Fig. 1-2*D*). Thus, learning is dependent on the presence of stimulus and is not an artifact of some unknown confound.

In the TPSD task, the P and NP stimuli had the same number of intratrial stimuli; because each was a different duration, the total durations of the sequences were different (P = 1.4 s; NP = 4.2 s). To control for this potential confound, we subjected a separate cohort of mice (*n* = 2) to a modified task (TPSD_mod_; Extended Data Fig. 1-3*A*). Additionally, we inverted the stimuli such that the longer intratrial stimulus was the P and the shorter was the NP to ensure that learning was not dependent on a specific duration or simply the shorter of the two durations. Mice learned the task in a mean of eight sessions, with most improvement occurring in CRr (Extended Data Fig. 1-3*C–E*). Licking profiles additionally verified learning with licking ramping in predictability before the water reward in learned sessions but remaining at chance level in naive sessions (Extended Data Fig. 1-3*B*,*F–I*). We performed a modified P/NP stimulus ratio as before in which the P/NP stimulus ratio in the TPSD_mod_ task (7:3) was changed to 6:4 to rule out artifacts of experimental design. Performance remained similar to learned sessions (Extended Data Fig. 1-4*A–D*). Control sessions were then run in which the monitor and speakers were turned off; performance decreased as before in the original paradigm to chance levels (Extended Data Fig. 1-4*E–H*).

The TPSD task is a go/no-go task composed of two sequences of synchronous audiovisual stimuli (Fig. 1*B*). Visual stimuli are 90° drifting sinusoidal gratings and are accompanied by a synchronous 5 kHz tone at 80 dB. Within each sequence, four stimuli are presented that differ only in temporality. Our preferred sequence is composed of four stimuli of 200 ms; our nonpreferred sequence is composed of four stimuli of 900 ms.

Each set of the sequences is separated by a 200 ms period of silence accompanied by a gray screen. A water reward is dispensed at 1.2 s and remains available until 2 s, at which time it is sucked away by a vacuum. A custom-built lickport (UCLA engineering) dispensed water, vacuumed it, and recorded licking via breaks in an infrared beam. Breaks were recorded at 250 Hz. The window in which the licking of mice counts toward a response is 1–2 s in both stimuli. A time-out period (6.5–8 s), in which the monitor shows a black screen and there is silence, is instituted if the mouse incorrectly responds. The first session was composed of 250 trials, and subsequent days of 350. Depending on the stimulus presented, the behavioral response of the animal was characterized as “Hit,” “Miss,” “Correct Rejection” (CR) or “False Alarm” (FA; Fig. 1*B*). An incorrect response resulted in the time-out period.

To expedite learning, we set the preferred (P)/nonpreferred (NP) stimuli ratio to 70:30 as we found that mice are more prone to licking (providing a “yes” response) than to inhibiting licking (providing a “no” response). We additionally instituted an individualized lick rate threshold to encourage learning as we found that lick rates differed significantly from mouse to mouse. Licking thresholds were calculated from lick rates for mice and shows no significant correlation between licking thresholds and learning rates (Pearson’s correlation coefficient, *r* = 0.4684; *p* = 0.3012). This indicates that the individualized lick rate threshold was used as a learning aid to complete the task and did not affect their learning rates or their reliance on the stimulus for task completion. To confirm that mice learned rather than took advantage of the biased 70:30 preferred to nonpreferred trial ratio, we tested mice for two additional sessions using a 60:40 ratio of preferred to nonpreferred stimuli (Fig. 1). We retain a greater number of preferred stimuli as the total time mice encounter preferred stimuli is less than that of encountering nonpreferred stimuli within a 60:40 trial session (294 vs 588 s, respectively). Following this, mice performed a control task, during which visual and auditory stimuli were not presented. Our data show that mice did not retain learned performance, indicating that they relied on the sensory stimuli for task completion (Fig. 2).

We additionally performed experiments on mice (*n* = 2) using a modified paradigm of TPSD (TPSD_mod_) in which the longer duration was the preferred stimulus and the shorter was the nonpreferred (Figs. 1-3). We modified the paradigm to also have the same total time between the preferred and nonpreferred stimuli (2.6 s). This paradigm entailed either three or seven synchronous audiovisual stimuli separated by 0.2 s gray screens, in which there was no sound. The preferred stimulus was three intratrial stimuli of 733 ms; the nonpreferred stimulus was seven intratrial stimuli of 200 ms. Water was dispensed at 2.3 s in the preferred stimulus. The period in which lick counted toward a decision was 2–3.2 s. Water remained available to the mice until 3.2 s. Like in the original paradigm, lick rate thresholds were individualized to mice.

10.1523/ENEURO.0047-23.2023.f3-1 Figure 3-1 V1 neural activity changes with learning (*n* = 5). ***A***, Mean spiking activity in naive and learned sessions by stimulus type in preferred period. Shaded areas represent 95% confidence intervals. ***B***, Calcium-dependent facilitation (CDF) of maximum spiking in naive and learned sessions by stimulus type in preferred stimulus period [KS tests with Bonferroni correction: α = 0.0083; preferred naive (PN) vs preferred learned (PL): *D*_(0.1197)_, *p* = 0.0016; PN vs nonpreferred naive (NPN): *D*_(0.0795)_, *p* = 0.0731; PN vs nonpreferred learned (NPL): *D*_(0.1186)_, *p* = 6.36 × 10^−8^; PL vs NPN: *D*_(0.1376)_, *p* = 1.68 × 10^−4^; PL vs NPL: *D*_(0.1469)_, *p* = 7.79 × 10^−5^; NPN vs NPL: *D*_(0.225)_, *p* = 2.49 × 10^−11^). ***C***, Mean spiking activity in naive and learned sessions by stimulus type in nonpreferred period. Shaded areas represent 95% confidence intervals. ***D***, CDF of maximum spiking in naive and learned sessions by stimulus type in nonpreferred stimulus period (KS test: *D*_(0.3766)_, *p* = 5.68 × 10^−31^). Download Figure 3-1, EPS file.

10.1523/ENEURO.0047-23.2023.f3-2 Figure 3-2 V1 neural activity is successively suppressed in learned sessions (*n* = 5). ***A***, Mean spiking activity in naive session by trial outcome in nonpreferred period. Shaded areas represent 95% confidence intervals. ***B***, Mean spiking activity in learned session by trial outcome in nonpreferred period. Shaded areas represent 95% confidence intervals. Download Figure 3-2, EPS file.

10.1523/ENEURO.0047-23.2023.f3-3 Figure 3-3 Lick-modulated cells show differential activity based on trial outcome (*n* = 4). ***A***, Spike-sorted mean activity of all lick-modulated cells, all trials, in learned sessions. ***B***, Mean spiking activity of lick-modulated cells in learned sessions by trial outcome in preferred period. ***C***, CDF of maximum spiking of lick-modulated cells in learned sessions by trial outcome in preferred stimulus period (KS tests with Bonferroni correction: α = 0.0167; Hit vs CR: *D*_(0.169)_, *p* = 0.2388; Hit vs FA: *D*_(0.169)_, *p* = 0.2388; CR vs FA: *D*_(0.0704)_, *p* = 0.9928). ***D***, Mean spiking activity of lick-modulated cells in learned sessions by trial outcome in nonpreferred period. ***E***, CDF of maximum spiking of lick-modulated cells in learned sessions by trial outcome in nonpreferred stimulus period (KS test: *D*_(0.1831)_, *p* = 0.1654). ***F***, SVM predictability between licking and neural activity of lick-modulated cells is comparable, indicating successful extraction of lick-modulated cells. Predictor is, respectively, licking per 0.067s bins and lick-modulated cell neural activity per 0.067 s bins in learned sessions. Outcome is stimulus type. Download Figure 3-3, EPS file.

10.1523/ENEURO.0047-23.2023.f3-4 Figure 3-4 SVM performance as a function of time comparing licking, lick-modulated cell neural activity, and non-lick-modulated cell neural activity in learned sessions (*n* = 5). ***A***, SVM predictability comparing Hit and CR trials. ***B***, SVM predictability comparing Hit and FA trials. ***C***, SVM predictability comparing CR and FA trials. ***D***, Control for ***A***. ***E***, Control for ***B***. ***F***, Control for ***C***. Download Figure 3-4, EPS file.

**Figure 5.**
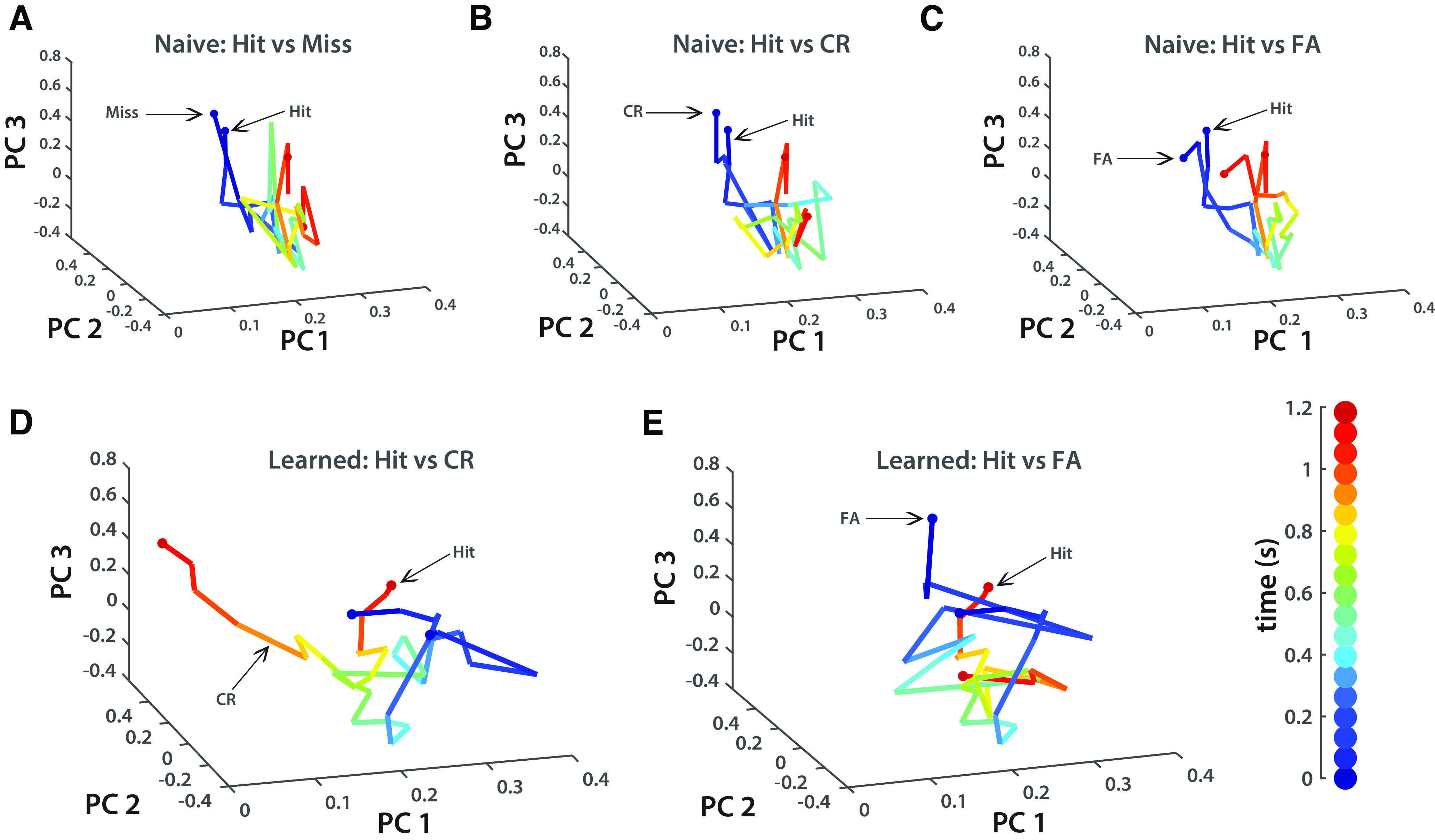
Neural trajectories diverge only in correct trials in learned sessions (*n* = 5). ***A***, Neural trajectories of Hit and Miss trials, naive sessions. ***B***, Neural trajectories of Hit and CR trials, naive sessions. ***C***, Neural trajectories of Hit and FA trials, naive sessions. ***D***, Neural trajectories of Hit and CR trials, learned sessions. ***E***, Neural trajectories of Hit and FA trials, learned sessions. Vertical bar at bottom right shows color coding of time through trials.

10.1523/ENEURO.0047-23.2023.f3-5 Figure 3-5 SVM cell selectivity is time and stimulus dependent. ***A***, Heatmaps of sorted cell selectivity as a function of time in small number cell selection groups. Bars represent how many times a given cell was selected at a given time as a proportion of the total number of possible selections (e.g., when 2 cells were selected, in a given time bin 2000 total selections could be made due to 1000 bootstrap iterations; therefore, if a cell were selected in every iteration, it would account for 50% of the total selections for that time bin). ***B***, Heatmaps of sorted cell selectivity as a function of time in large number cell selection groups. Due to more cells being selected in larger number cell groups, some cells may be selected whether they are or are not predictive (e.g., in “Learned: 100 cells,” one mouse had exactly 100 cells, therefore, each cell was selected in each iteration regardless of how informative it was). Download Figure 3-5, EPS file.

Custom-written routines and Psychtoolbox in MATLAB were used to present the visual stimuli, to trigger the lickport to dispense and retract water, and to acquire data.

### Cranial window surgery

Craniotomies were performed at 6–8 weeks. Before surgery, mice were given dexamethasone (0.2 mg/kg, i.p.) and carprofen (5 mg/kg, s.c.). Mice were anesthetized with isoflurane (induction, 5%; maintenance via nose cone, 1.5–2%) and placed in a stereotaxic frame. Under sterile conditions, a 4.5-mm-diameter craniotomy was drilled over the right V1 and covered with a 5 mm glass coverslip. Before securing the cranial window with a coverslip, we injected 60–100 nl of pGP-AAV-syn-jGCaMP7f-WPRE. A custom U-shaped aluminum bar was attached to the skull with dental cement to restrain the head of the animal during behavior and calcium imaging. For 2 d following surgery, mice were given dexamethasone (0.2 mg/kg) daily.

### Viral constructs

pGP-AAV-syn-jGCaMP7f-WPRE were purchased from Addgene and diluted to a working titer of 2e^13^ with 1% filtered Fast Green FCF dye (Thermo Fisher Scientific).

### *In vivo* two-photon calcium imaging

Calcium imaging was performed on a Scientifica two-photon microscope equipped with a Chameleon Ultra II Ti:sapphire laser (Coherent), resonant scanning mirrors (Cambridge Technologies), a 20× objective (1.05 numerical aperture; Olympus), multialkali photmultiplier tubes (catalog #R3896, Hamamatsu) and ScanImage software (Pologruto et al., 2003). Before calcium imaging, head-restrained mice were habituated to a soundproof chamber and allowed to run freely on a polystyrene ball (Figs. 1*A*, 3*A*). Visually evoked responses of L-2/3 pyramidal (Pyr) cells from V1 were recorded at 15 Hz in 1 field of view (FOV). Each FOV consisted of a mean of 108 Pyr cells (SD = 39.2). In each animal, imaging was performed at 150–250 μm.

### Data analysis

#### Discriminability index and CR and Hit rates

The discriminability index (*d′*) was calculated using the MATLAB function *norminv*, which returns the inverse of the normal cumulative distribution function, as follows:

$$d\prime \text{ = norminv}\left(\text{fraction of hits}\right)-\text{norminv}(\text{fraction of FAs}).$$

If either rate reached 100% or 0%, we arbitrarily changed the value to either 99% or 1%, respectively. We did this to avoid generating *z* scores of infinity that would inaccurately characterize the performance of the mice.

The *d′* values of the best 150 trials were selected by a sliding 150 trial window; the highest value was then selected. CR rates (CRrs) and Hit rates (Hrs) use the same best 150 trial interval.

#### Licking thresholds

Licking thresholds for each mouse was determined by using the average licking in the last Pretrial session minus 1 SD.

#### Licking probabilities

Probabilities were taken by binning licks per 0.1 s window per trial per mouse. We then averaged the probability per time of each mouse to generate a distribution of probabilities based on trial session, stimulus type, and trial outcome. We use the best 150 trials from each day and each mouse as determined by the *d′* value.

#### Data analysis for calcium imaging

Calcium-imaging data were analyzed using suite2p (Pachitariu et al., 2017) and custom-written MATLAB routines. All data were then processed using suite2p for image registration, region of interest (ROI) detection, cell labeling, and calcium signal extraction with neuropil correction. Once suite2P had performed a rigid and nonrigid registration and then detected ROIs using a classifier, we manually selected cells using visual inspection of ROIs and fluorescence traces to ensure the cells were healthy. We then used the deconvolved spikes determined by suite2p in our subsequent analysis that used custom-written MATLAB scripts.

#### Mean network activity

We performed a bootstrap of 1000 iterations per mouse to select average activity patterns. Putative spikes were composed of either on or off times (0s or 1s). We then composed a grand distribution and used average network activity (Fig. 3*F*,*H*, Extended Data Fig. 3-1*A*,*C*). Shaded areas represent 95% confidence intervals of each respective activity curve.

#### Correlation of mean network activity with stimuli

Pearson’s correlations were calculated using each bootstrapped iteration of mean network activity and a separate matrix of 0s and 1s, with 0s representing stimulus off periods and 1s representing stimulus on periods.

#### Time-sorted heatmaps

Heatmaps featuring sorted activity (Fig. 3*E*, Extended Data Fig. 6-1) were sorted using the maximum value over a given time course per unit. Units were then displayed such that cells having a maximum value at time *t* were placed together; each successive grouping of cells at *t + 1* was placed below the previous value *t*.

#### Lick-modulated cells

Lick-modulated cells were determined by using a bootstrapped support vector machine (SVM; see below for SVM methods). Hit and FA trials were compared with CR and Miss trials within the response period. The difference between these two predictors within this period should only be whether there was licking or not licking, as stimuli and the presence or absence of reward differ within predictors. The *sequentialfs* function in MATLAB, a sequential forward feature selection function, was used to identify 20 cells that contained the most predictive information per mouse per time bin per session. Upon completion, each time a cell was selected, it received a score based on the *z*-scored accuracy of the prediction (e.g., <50% accuracy resulted in a negative score, >50% accuracy resulted in a positive score, 50% accuracy resulted in a 0 score). All scores were then summed. The total scores of cells were then correlated with the number of times they were selected per mouse per session. Correlations that were positive and significant (α = 0.05), indicative of reliable predictability, were admitted. Cells that were >1 SD from the mean of the total scores were then selected as lick-modulated cells; 16.83 ± 8.5 lick-modulated cells were found per mouse. One mouse was found to have no lick-modulated cells.

#### Neural trajectories

We averaged the activity of each cell over each of its trials based on output (Hit, Miss, CR, FA) and then ran a principal component analysis using the MATLAB function *pca*. Trajectories of the first three components were plotted. The variance explained as follows: mean = 75.5, SD = 11.9.

#### SVM

We used the SVM available in the MATLAB Machine Learning and Deep Learning toolbox via the function *fitcsvm*. We used a radial basis function as the kernel. Eighty percent of our data were applied to training the machine, and 20% were applied to testing it. Instead of training one machine, we developed a strategy wherein we performed a bootstrapped SVM per time per mouse. This allowed us to generate a distribution of accuracy percentages per time such that we could locate critical times of difference during stimulus presentation. Ten thousand machines were generated per time per mouse for the licking predictor and then were averaged as one grand distribution. One thousand machines were generated per time per mouse for the imaging predictor and then were averaged as one grand distribution. The fewer number of machines for the imaging predictor was because of computational constraints. The licking predictor consisted of binning licks per 0.067 s window per trial per mouse with either the stimulus type (preferred or nonpreferred) or trial outcome (Hit, Miss, CR, FA) as the outcome. The imaging predictor was the activity of the network with either the stimulus type (preferred or nonpreferred) or trial outcome (Hit, Miss, CR, FA) as the outcome in 0.067 s time bins. For a given mouse, the putative spiking activity of each cell was used as a feature space per a given time. With our licking data, we performed no pretraining optimization as we essentially were testing the accuracy of individual features (i.e., time bins of licking).

**Figure 6.**
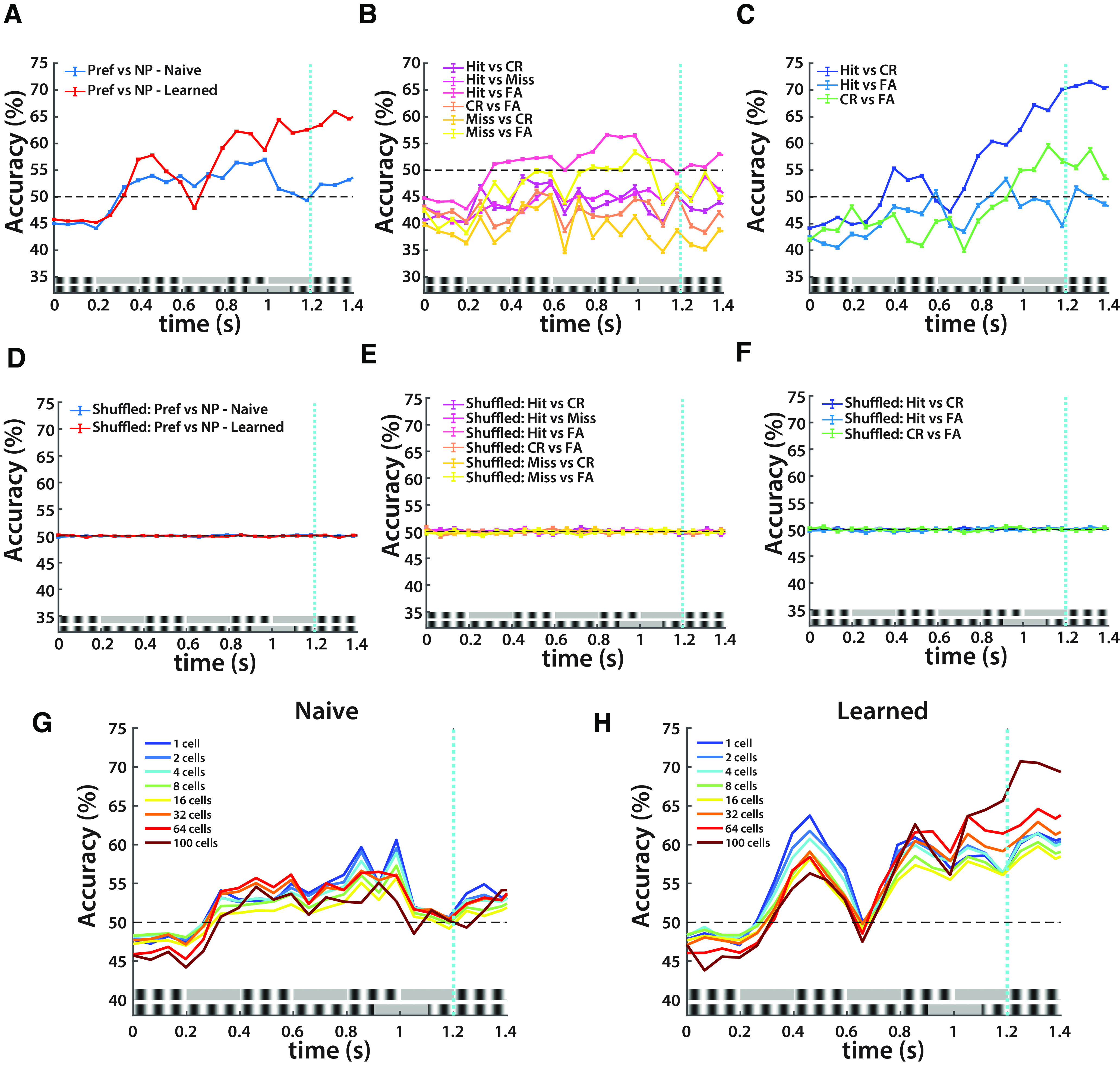
SVM performance as a function of time (*n* = 5). ***A***, Spiking activities per mouse per trial in naive and learned sessions were predictors, and outcome was the stimulus type. ***B***, Spiking activity per mouse per trial in naive sessions was the predictor, and outcome was the trial outcome (Hit, Miss, CR, FA). ***C***, Spiking activity per mouse per trial in learned sessions was the predictor, and outcome was the trial outcome (Hit, CR, FA). Miss trials were again omitted because of their small sample size. ***D***, Control for ***A***. Spiking activities per mouse per trial in naive and learned sessions were predictors, and outcome was the randomly shuffled stimulus type. ***E***, Control for ***B***. Spiking activities per mouse per trial in naive sessions was the predictor, outcome was the randomly shuffled trial outcome. ***F***, Control for ***C***. Spiking activities per mouse per trial in learned sessions were predictors, and outcome was the randomly shuffled trial outcome. ***G***, SVM decoding accuracy of spiking activity in naive sessions with varying numbers of cells selected. A forward sequential feature selection algorithm was used to select predictive cells (see Materials and Methods). ***H***, Same as ***G***, but in learned sessions. Extended Data Figure 6-1 provides evidence for cell selectivity during learning.

We performed optimization procedures on our neural data, however. We performed a fivefold cross-validation and used the built-in Bayesian optimizer in MATLAB (*bayesopt* function) to tune the hyperparameters (see Fig. 6*A–F*, all non-lick-modulated cells in the network as features). We also performed a feature selection procedure wherein we ran the SVM as done previously but by selecting a given number of cells as features (see Fig. 6*G*,*H*). This again entailed using the *sequentialfs* function in MATLAB to find the most predictive cells per a given interval. As an example, when we chose four cells, at each time bin for each mouse, the feature selection algorithm chose four cells that were most representative of the difference between two categories, which were then used to predict the difference between the two categories. Thus, there was a distribution of 4000 cells that were selected for that time point (4 cells by 1000 machines) and 1000 accuracy percentages (1000 machines). These machine accuracies were then averaged (see Fig. 6*G*,*H*), and the distribution of cells was sorted by time (Extended Data Fig. 6-1) to show when a given cell was most likely to be selected.

Each set of trials we performed with the SVM included all trials so as to have the most robust dataset possible. All uses of the SVM were accompanied by control trials in which outcomes were randomly shuffled.

### Statistical analyses

Statistical analysis of normality (Lilliefors test) was performed on each dataset, and, depending on whether the data significantly deviated from normality (*p* < 0.05) or did not deviate from normality (*p* > 0.05), appropriate nonparametric or parametric tests were performed. The statistical tests performed are mentioned in the text and the legends. For parametric two-group analyses, a Student’s *t* test (paired or unpaired) was used; for parametric multigroup analyses, a one-way ANOVA was used. For nonparametric tests, we used the following: Wilcoxon rank-sum test (two groups), Kolmogorov–Smirnov test (KS; two groups), and Kruskal–Wallis test (multigroup). When multiple two-group tests were performed, a Bonferroni correction was applied to readjust the α value. In the figures, significance levels are represented with the following convention: *p < α; **p < α/10, ***p < α/100. α Values are 0.05 unless otherwise specified. In all of the figures, we plot either the SEM or 95% confidence intervals. Graphs show either individual data points from each animal or group means (averaged over different mice) superimposed on individual data points.

### Exclusion of mice

Five WT mice were excluded from the data because the mice lost >25% body weight (a criterion we established a priori). This had adverse effects on their health that was manifested in listlessness, reduced grooming, interaction with cage mates, and, occasionally, seizures.

### Data availability

All the analyzed data reported in this study are available from the corresponding author on request. All code, including the SVM analysis used in this article, is available from the corresponding author on request.

## Results

### Mice learn to perform a multimodal temporal pattern sensory discrimination task

To examine temporal pattern learning, we have designed a novel go/no-go TPSD task (see Materials and Methods). We test our paradigm in mice as they are a robust animal model for temporally and spatially fine recording methods and for cell type-specific tagging and manipulation. Awake, head-restrained young adult mice (2–3 months of age) are habituated to run on a polystyrene ball treadmill while they perform the TPSD paradigm. Water-deprived mice are presented with two audiovisual temporal patterns (preferred and nonpreferred), as shown in the schematic in Figure 1*B*. Each pattern consists of four AV stimuli, where each AV stimulus lasts either 0.2 or 0.9 s and is separated by a 0.2 s gray screen. The visual stimulus consists of a drifting sinusoidal 90° grating, and the auditory stimulus consists of a 5 kHz tone. Both auditory and visual stimuli are presented concurrently; therefore, the stimuli are audiovisual. The temporal pattern with 0.2 s AV stimuli is associated with a water reward (preferred pattern), and the temporal pattern with 0.9 s AV stimuli is not (nonpreferred pattern; Fig. 1*B*). We quantified the performance of mice using a *d′* value in which *d′* = 2 was set as a learning threshold (Fig. 1*C*). Mice learn to preferentially lick to the preferred pattern and to withhold licking for the nonpreferred pattern (12.13 ± 3.52 sessions to learn; *n* = 8; one-way ANOVA, *F*_(1,16)_ = 5.45, *p* = 1.23 × 10^−7^). A positive *d′* value of 0.5 on session 1 likely resulted from mice learning to associate stimulus with reward in the pretrial task before the TPSD task. During the pretrial task, mice experience only the preferred stimulus and every trial is rewarded. This allows mice to learn to lick reliably (>80% licking) and learn the task structure–association of stimulus with water reward (see Materials and Methods). This pretrial task is similar to previous studies (Goel et al., 2018) and is a common strategy used in behavior assays (Guo et al., 2014).

Similar to other go/no-go tasks, to aid learning, we use a 7:3 preferred to nonpreferred stimulus ratio in the main task, which can artificially amplify the effect of Hit rate on the *d′* value of the mice. To confirm that learning is not simply a biased feature of the differential preferred/nonpreferred ratio and to examine the decision strategy of mice between learned and naive days, we compared Hrs with CRrs (Fig. 1*D*,*E*). Mice improved performance primarily by improving their CRrs in which the CRr changed from negative to positive (Fig. 1*E*; *n* = 8; two-tailed, paired-sample Student’s *t* test, *t*_(14)_ = 4.46, *p* = 5.3 × 10^−4^). Although significant, we find that the Hr of mice remains relatively unchanged across sessions (Fig. 1*E*; *n* = 8; two-tailed, paired-sample Student’s *t* test, *t*_(14)_ = 4.42, *p* = 5.85 × 10^−4^) but that their ability to inhibit licking increases across sessions, indicated by a positive CRr in learned sessions.

Licking profiles that accompany learning were more refined in expert mice (Fig. 2). We quantified the probabilities of the licking by the mice based on session day (naive vs learned), stimulus type (preferred vs nonpreferred), and trial outcome (Hit, Miss, CR, FA) as a function of time. We find that on learned days licking to the preferred stimulus is enhanced, while licking to nonpreferred stimuli dramatically decreases before the water reward, indicative of a learned response (Fig. 2*B*). The most robust change in licking occurred in the nonpreferred stimulus in which mice peak in their lick probabilities in CR trials before the onset of the water reward in learned days (Fig. 2*D*).

### Licking profiles in learned mice predict stimulus type

To causally establish that licking is both (1) a viable measure of performance and (2) demonstrates differential learning between sessions, we developed a bootstrapped SVM, a type of binary classifier. We run our SVM 10,000 times within 0.067 s time bins using licking within a trial as the predictor and the stimulus of that trial as the outcome (see Materials and Methods). This allows us to generate a distribution of correctly predicted outcomes per time bin per mouse, which are then compared with a randomly shuffled control. We find that there is little predictability beyond chance in naive days with somewhat greater predictability following the water reward, likely attributable to increased licking at their chance encounter with the water reward (Fig. 2*E*). On learned days, licking becomes predictive beyond chance at 0.7 s and then accelerates beginning at 0.8 s. This suggests that mice relied on stimulus information to make a decision rather than on the presence or absence of the water reward. The high performance of the SVMs before the water reward in learned sessions establishes that mice indeed learn to discriminate temporal patterns. In addition, it identifies the decision period at ∼0.7–0.8 s.

### Learning is not an artifact of behavior design

Following learning, mice underwent the following two additional protocols: (1) performing the same paradigm with a 6:4 P/NP stimulus ratio; and (2) performing the same paradigm without any visual or auditory input as a control (Control). We performed the 6:4 P/NP stimulus ratio task to confirm that learning was not a feature of the differential P/NP stimulus ratio of 7:3 in the main task, and the Control task to confirm that learning was stimulus dependent.

In the 6:4 P/NP stimulus ratio task, the *d′* values of mice for CRr and Hr were not significantly different from the main task (Extended Data Fig. 1-1*A*). Licking probabilities were similar as well, albeit there was less overall licking in the P/NP stimulus ratio of 6:4 task than in the main task (Extended Data Fig. 1-1*B*,*C*). We again used licking to predict stimulus type and found that predictability was maintained nearly identically in the P/NP stimulus ratio of 6:4 task (Extended Data Fig. 1-1*D*). These results confirm that learning was not a result of the P/NP stimulus ratio of the main task.

### Pyramidal cell dynamics in primary visual cortex accompany temporal pattern learning

A previous published study using sensory cortical organotypic slice cultures (Goel and Buonomano, 2016) found that information about stimulus duration is encoded in a change in pyramidal cell activity wherein the neural activity is refined to represent the learned interval. We predicted that a similar emergent neural activity contributed to learning temporal patterns *in vivo*. Therefore, to examine the pyramidal cell dynamics that are associated with TPSD, we performed two-photon calcium imaging in V1 to provide a real-time assay of neural activity during TPSD. Studies have shown that auditory inputs strongly influence neural responses in primary visual cortex (McIntosh et al., 1998; Zangenehpour and Zatorre, 2010; Deneux et al., 2019; Garner and Keller, 2022) and audiovisual stimuli evoke multimodal plasticity in V1 (Morrell, 1972; Petro et al., 2017), thus justifying V1 dynamics as a locus of change accompanying learning on the TPSD task.

We recorded from V1 L-2/3 using two-photon calcium imaging and jGCaMP7f (half-rise time = 27 ± 2 ms) with a synapsin promotor via an adeno-associated virus (AAV) vector (Dana et al., 2019; Fig. 3*A–C*). This indicator has been used by numerous published studies and is routinely used by researchers performing calcium imaging during behavior because of its enhanced signal-to-noise ratio and fast rise-time kinetics. Mounting evidence suggests that movement-related activity accounts for a considerable amount of variance in neural recordings, including in primary sensory areas (Zagha et al., 2022). Further, timing and movement are linked, and therefore neural dynamics that encode time information may also code for licking. To exclusively examine neural codes for the temporal structure of the pattern, following recording we identified lick-modulated cells and removed them from subsequent analyses of neural data (see Materials and Methods; Extended Data Fig. 3-3) to distinguish sensory activity from motor and/or decision-related activity.

We find that in mice (*n* = 5) during the TPSD task, there are time-dependent changes in mean network activity between naive and learned sessions. Naive sessions do not show temporal structure in either P or NP stimuli throughout the period of either (Extended Data Fig. 3-1*A*,*C*). Learned sessions show changes in activity to both stimuli. Activity is correlated until ∼0.7 s, at which there remains sustained activity in the preferred condition and suppressed activity in the NP condition (Extended Data Fig. 3-1*A*,*C*). Additionally, activity in the preferred condition is temporally coordinated with the preferred stimulus, peaking at times of intrastimulus presentation. Activity in the nonpreferred condition is likewise time locked to the preferred stimulus until 0.7 s. We suspect that the network predictively codes the preferred stimulus in both conditions, but that it is successively suppressed as it encounters the nonpreferred stimulus. Cumulative distributions of maximum spiking support this hypothesis as NP maximum spiking is significantly left shifted in learned sessions compared with naive sessions (Extended Data Fig. 3-1*B*,*D*).

Furthermore, network activity in naive sessions does not distinguish trial outcome (Hit, Miss, CR, FA; Fig. 3*E*,*F*, Extended Data Fig. 3-2*A*). In learned sessions, network activity indexes both stimuli and trial outcome (Fig. 3*G*,*H*, Extended Data Fig. 3-2*B*). In learned Hit trials, activity is temporally coordinated with the preferred stimulus (Fig. 3*H*). Hit learned session activity is the only condition in which activity is positively and significantly correlated with the preferred stimulus (learned Hit: (*r* = 0.0134, *p* = 8.33 × 10^−6^; learned CR: *r* = –0.004, *p* = 0.1892; learned FA: *r* = 0.0077, *p* = 0.0112; naive Hit: *r* = –0.008, *p* = 0.0073; naive Miss: *r* = –0.0123, *p* = 1.76 × 10^−12^; naive CR: *r* = −5, *p* = 0.8; naive FA: *r* = -0.021, *p* = 1.31 × 10^−12^). CR trials show suppression of activity at ∼0.7 s, whereas FA trials show delayed suppression, likely leading to the incorrect response (Fig. 3*G*,*H*, Extended Data Fig. 3-2*B*). Delayed suppression in learned FA trials lasts until the preferred stimulus period ends at 1.4 s. We suspect that FA trial activity is predictively coded as the preferred stimulus, leading to sustained activity. CR trial activity is coded as the preferred stimulus until 0.7 s, at which point broad inhibitory activity likely suppresses the network.

We examined when cells were mostly likely to fire in naive and learned sessions. In naive sessions, maximum spiking was significantly different save for between Hits and FAs, and Misses and CRs, respectively, which indicates that network activity was not driven exclusively by sensory discrimination (Fig. 4*A*). Learned sessions showed significant differences between Hits and CRs, and Hits and FAs, respectively, which indicates that temporal features generate distinct network activity (Fig. 4*B*). Additionally, learned session CR and FA trials were significantly left shifted from naive session CR and FA trials, with learned CR trials the most left shifted (Fig. 4*C*). This demonstrates that the network in naive sessions was active throughout the NP stimulus period, whereas in learned sessions, the network had been tuned to the preferred stimulus and therefore suppressed activity in nonrelevant stimuli. Additionally, the level of suppression of activity in the NP stimulus indexes correct or incorrect responses.

### Visual cortical neural dynamics contain temporal information

As previously discussed, we suspected that learning would be enabled through distinct patterns of network dynamics. That is, the amount of difference in neural trajectories through state space should index learning. Indeed, on naive days in which performance is poor, there is little difference in network activity regardless of stimulus type or trial outcome (Fig. 5*A–C*). In learned sessions, the trajectory of the network shows divergence between Hit and CR trials (Fig. 5*D*), but shows clustering in Hit and FA trials (Fig. 5*E*). Notably, the greatest divergence between Hit and CR trajectories in learned sessions begins at ∼0.7 s, the period at which licking also began to be most predictive of stimulus type in learned sessions. We suspect that this divergence does not exist between Hit and FA trials as FA trials predictively code the preferred stimulus.

However, an outstanding question is whether network divergence causes a decision to be made or whether the decision is made elsewhere, which then leads to feedback release or inhibition (I) of the network. We addressed this by using our bootstrapped SVM (0.067 s time bins, 1000 iterations/bin) to predict stimulus and trial outcome from neural activity. We found that network activity accurately predicts stimuli in learned sessions and moderately so in naive sessions (Fig. 6*A*). In naive sessions, there is little predictability, suggesting that the network is not differentially tuned to stimuli or trial outcome (Fig. 6*B*). In learned sessions, there is high predictability between Hit and CR trials, moderate predictability between CR and FA trials, and no predictability between Hit and FA trials (Fig. 6*C*). The respective predictability profiles of Hit versus CR and Hit versus FA in learned sessions accord with the analysis of neural trajectories in which CR trials diverge from Hit trials, but FA trials do not (Fig. 5*D*,*E*). Because Hit and FA trials generate the same network response, evinced by predictability at chance levels, it is likely that FA trials predictively encode the preferred stimulus. Thus, in learned sessions, accurate encoding of stimuli directly contributes to trial outcome.

10.1523/ENEURO.0047-23.2023.f6-1 Figure 6-1 V1 L-2/3 encodes temporal information in flipped paradigm in learned sessions (*n* = 2). ***A***, Heatmaps of spike-sorted activity in mice in naive sessions based on trial outcome. Lick cells were removed prior to the analysis of neural activity in both sessions as was done in the original TPSD paradigm analysis. ***B***, Heatmaps of spike-sorted activity in mice in learned sessions based on trial outcome. ***C***, Mean network spiking activity based on stimulus or trial outcome and session day. CR trials in the naive sessions and Miss trials in the learned session were removed due to exceedingly small samples. ***D***, Cumulative distributions of maximum spiking activity based on stimulus or trial outcome and session day. CR trials in the naive session and Miss trials in the learned session were removed due to exceedingly small samples. ***E***, SVM predicts stimulus from neural activity in learned sessions but remains at chance level in naive sessions. Download Figure 6-1, EPS file.

Not surprisingly, the neural dynamics of lick-modulated cells contained information about the trial outcome (Extended Data Fig. 3-3). We further verified this finding by comparing licking predictability with neural predictability in learned sessions (Extended Data Fig. 3-4). In Hit versus CR trials, neural predictability first rises above chance in nonlick cells at ∼0.3 s and then ramps at 0.7 s; predictability of lick-modulated cells and licking, however, occurs later at 0.7 s (Extended Data Fig. 3-4*A*). In Hit versus FA trials, there is no predictability in nonlick cells, lick-modulated cells, or licking (Extended Data Fig. 3-4*B*). These results indicate that it is necessary for the network to accurately encode temporal information before making a decision, and thus implicates sensory-driven activity in learning and trial outcome.

We additionally recorded from V1 L-2/3 in a separate cohort of mice (*n* = 2) using our TPSD_mod_ paradigm to ensure that learning was not dependent on potential artifacts within our original paradigm (Figs. 1-3). Notably, we found the network dynamics tune to the longer preferred stimulus as indicated by a right-shifted distribution in maximum spiking in Hit trials in learned sessions compared with naive sessions (Extended Data Fig. 3-5*D*). CR maximum spiking was left shifted, indicating suppression of nonrelevant stimuli similar to that seen in learned sessions in the original paradigm (Extended Data Fig. 3-5*C*,*D*). Additionally, the decoder accurately predicted stimulus type from learning in learned sessions but not in naive sessions (Extended Data Fig. 3-5*E*).

### Temporal information is encoded by intrinsic mechanisms in V1

Based on the results of the SVM using the entire network, we next characterized the dynamics by which V1 encoded temporal information. It has been shown previously that temporal information can be encoded in a variety of neural mechanisms including linear ramping, high-dimensional dynamics, and a combination of oscillators (Bakhurin et al., 2017; Zhou et al., 2020). Here we specifically addressed whether temporal coding in the TPSD task relies on dedicated or intrinsic mechanisms. We tested this by using neural data to predict stimulus outcome in learned and naive sessions as before, but by using a forward sequential feature selection algorithm for various numbers of cells, with each cell for a given mouse representing a feature. First, if temporal information is encoded in specialized functionally connected cells or circuits (i.e., through dedicated mechanisms), we predict that we would see high predictability of stimuli using a small subset of cells in both naive and learned sessions. Additionally, we predict that the same cells would be selected throughout the stimulus period as they contain a unique ability to represent temporal information. If, however, temporal information was encoded through intrinsic mechanisms, we predict that cell selection would vary through the stimulus period, that greater numbers of cells would provide higher predictability, and that predictability would emerge over learning likely through refinement of the network.

We find that there is only high predictability in learned sessions and that cells that are selected vary over the stimulus period, indicating that in V1 timing is achieved without dedicated timing mechanisms but rather through changing patterns of intrinsic neural dynamics (Fig. 6*G*,*H*, Extended Data Fig. 6-1). Additionally, predictability and selectivity in learned sessions is stimulus dependent. These results suggest that temporal information in V1 does not invariantly rely on dedicated mechanisms.

However, we find that systematically there is higher predictability earlier in the stimulus period in fewer cells in the learned sessions. The high early predictability ramps immediately following 0.2 s, which is the first period at which the P and NP stimuli differ. This result suggests that in V1, temporal information can be encoded by a small subset of cells. However, as the stimulus period continues, and a decision is reached before the arrival of water, there is ramping in predictability in all cell selections, and in greater numbers of cells there is systematically greater predictability. Notably, at 0.4–0.5 s, in which stimuli are visually and aurally the same, there remains high predictability that differentiates P and NP stimuli, which implicates temporal information as what is most saliently encoded in V1.

We suspect that a small subset of cells at ∼0.2 s indexes the temporal difference between stimuli whose activity then propagates throughout the network as the stimulus period continues and more sensory information is received. This leads to network-level tuning, which causes the network itself to be more predictive than a small subset of cells. However, as this ramping in network-level predictability occurs following the decision period, it is unknown whether this tuning is caused by the stimulus, by the early activity of a small subset of cells that generate a particular neural trajectory, or by top-down areas amplifying relevant functional populations in V1. Thus, a combination of distinct mechanisms may be responsible for timing within the stimulus period.

## Discussion

Using a go/no-go audiovisual timing task, we have demonstrated that mice learned to perform the TPSD task successfully, as assessed through an increase in the discriminability index and a refinement of licking profiles. Learned performance was attributable to changes in response to the nonpreferred temporal pattern in which licking was suppressed early into the stimulus period. Similar results were seen in neural activity in V1 L-2/3 in which activity was suppressed in the nonpreferred stimulus but was released in a temporally defined manner in the preferred stimulus. Whereas naive sessions showed decided overlap in neural trajectories, learned sessions showed trajectories that indexed stimulus type and trial outcome, suggesting that distinct functional populations developed with learning. Neural activity was also used to decode stimulus type and trial outcome in learned sessions but not in naive sessions, which indicates that V1 undergoes synaptic plasticity changes to support temporal learning. Additionally, we found that subsecond temporal encoding relies on intrinsic temporal mechanisms. Early decoding predictability using a small subset of cells suggests that the state the of entire network does not index temporal information but that it is contained within a few cells. As the stimulus period progresses, the dynamics of the entire network become more predictive than a small subset of cells, indicating that the network state does index elapsed time. However, how this transition occurs and whether it is achieved locally or via top-down inputs require further investigation.

Because of the complexity of understanding temporal processing, time has been categorized into distinct types such as sensory versus motor timing and interval versus pattern timing, as well as distinguishing between different timescale ranges (Paton and Buonomano, 2018). For example, many tasks in interval timing require the identification of isolated segments of time such as in waiting to cross a street or identifying a single musical note. While interval timing can be studied as its own entity, it is also important in pattern timing. Pattern timing is composed of intervals and contains a temporal structure (Paton and Buonomano, 2018). For instance, to understand language, one must recognize the overall prosody of speech, as well as the pauses between words. The timescale in which interval and pattern timing occur is on the order of tens of milliseconds to a few seconds, although it is unknown whether the neural mechanisms of interval and rhythmic timing are shared and whether intrinsic timing mechanisms that contribute to interval timing also contribute to learning of temporal patterns (Hardy and Buonomano, 2016). Although early theories of timing proposed centralized mechanisms dedicated entirely to temporal perception, it has since been established that different neural mechanisms are involved in processing time at different timescales (Paton and Buonomano, 2018). However, it has yet to be determined whether the mechanisms of temporal perception in the range of seconds and subseconds are distributed across brain regions or whether local networks within different regions can intrinsically encode time, albeit through a diversity of network dynamics (Zhou et al., 2020, 2022). Primary visual cortical circuits show robust plasticity to spatiotemporal features in stimuli and predict temporal associations (Chubykin et al., 2013; Gavornik and Bear, 2014a,b; Fiser et al., 2016; Garrett et al., 2020). Our data show robust V1 dynamics that encode temporal information about the experienced stimuli as shown by the accuracy of the decoder. As is the case in many behaviors, perturbation experiments are typically performed to establish causality between neural activity and behavior. However, using machine-learning algorithms to show a requirement of neural activity is increasingly used by many groups (Bakhurin et al., 2017; Zhou et al., 2020; Lazar et al., 2021; Toso et al., 2021). While not the same as a perturbation experiment, a bootstrapped SVM allowed us to determine whether neural activity contained information about stimulus type and trial type. Importantly, we find that information in the TPSD task is learned through intrinsic changes in V1 dynamics. Further, depending on the time during the trial duration, learned information about the temporal patterns was encoded in a small selection of cells indicative of “time cells,” a medium-sized selection indicative of oscillators, as well as a large selection of cells suggestive of a change in network state. In conclusion, our data show the contribution of multiple mechanisms that allow learning and representation of time intervals.

There is a growing consensus that movement-related and arousal-related signals as well as sensory and cognitive processes may be contained in the same evolving neural activity (Zagha et al., 2022). However, identifying and dissociating neural circuits and dynamics that contribute to sensory, motor, arousal, and other cognitive process is still a challenge. We used two strategies to address this challenge: (1) the design of the TPSD task, which consisted of a separation of stimulus onset from the response window, thus attributing the very early neural activity to sensory processes (temporal discrimination); and (2) we exclusively examined neural activity that contributed to encoding time by removing lick-modulated cells from our analysis.

Complex interplay of synaptic excitation (E) and I allows cortical neurons to adaptively respond to sensory stimuli, discriminate between stimuli, and integrate sensory inputs (Isaacson and Scanziani, 2011; Ferguson and Cardin, 2020). Converging evidence across many studies and model systems shows that selectivity to the interval of a stimulus duration is the result of dynamic shifts in E–I balance (Edwards et al., 2007; Kostarakos and Hedwig, 2012; Goel and Buonomano, 2016). Consistent with previous *in vitro* work in cortical slices (Goel and Buonomano, 2016), our data suggest that network suppression is one potential mechanism that drives learning. Encoding of intervals and patterns in sensory cortical circuits can result from changes in the E/I ratio at temporally defined periods (Goel and Buonomano, 2016) and by multiple interneuron populations (Cardin, 2018). V1 L-2/3 is composed primarily of Pyr cells, which are synapsed by parvalbumin (PV) cells at the cell body and axonal hillock (Gonchar and Burkhalter, 1997). Somatostatin (SST) cells synapse onto the dendrites of pyramidal cells, thus providing more fine-tuned inhibition. Both PV and SST cells have been implicated in short-term plasticity, which is one of the mechanisms proposed to drive subsecond sensory timing (Buonomano, 2000; Motanis et al., 2018; Seay et al., 2020). PV cells provide reliable inhibition within a short window, which can help to constrain pyramidal cell firing to temporally defined windows (Pouille and Scanziani, 2001; Cardin et al., 2010). We speculate that inhibition, driven by PV cell activity, which is broadly tuned because of their anatomic connectivity, can drive temporal encoding of rhythmic patterns. Indeed, in a recent study, PV neurons were shown to be important in mediating reward timing (Monk et al., 2020). However, SST cells also modulate cortical output. SST neurons not only provide dendritic pyramidal cell inhibition but also control PV cell output (Atallah et al., 2012). Therefore, a complex interaction between SST and PV cells determines the balance between somatic and dendritic inhibition on pyramidal cells (Xue et al., 2014), thus contributing to temporal encoding (Cardin, 2018). However, it is important to emphasize that circuits in V1 are not the only contributors to the TPSD task and that multiple areas such as auditory cortex, ACC, and other downstream areas are likely involved.

Our results thus far suggest that the emergence of complex neural dynamics in V1 accompanies temporal pattern learning. An important hallmark of learning is being sensitive to and remembering the temporal structure of events so that we can make predictions and guide our future decisions. As a result, it is not surprising that disruptions in timing and timed performance are associated with a number of neurologic disorders such as Parkinson’s disease, schizophrenia, and autism spectrum disorder. Our study opens the door to future studies probing the mechanistic substrates of excitation and inhibition that fine-tune temporal pattern learning and offers insights into translational studies of time perception.

## Synthesis

Reviewing Editor: Michaël Zugaro, CNRS, Collège de France, Inserm Decisions are customarily a result of the Reviewing Editor and the peer reviewers coming together and discussing their recommendations until a consensus is reached. When revisions are invited, a fact-based synthesis statement explaining their decision and outlining what is needed to prepare a revision will be listed below. The following reviewer(s) agreed to reveal their identity: NONE.

To examine how neural circuits in early visual cortex (V1) encode temporal information in sensory stimuli, this manuscript assesses the neural dynamics contributing to temporal discrimination in a go/no-go decision-making task in mice. The authors performed 2-photon Ca2+ imaging as mice learned a multi-modal (auditory and visual) temporal discrimination task using instrumental conditioning. The authors found that local network activity in V1 is somewhat predictive of stimuli and trial outcomes, and emerges with learning. Namely, activity was high and sustained for ‘hit’ and ‘false alarm’ trials, but was suppressed for ‘correct rejection’ trials. This differential activity pattern developed across training sessions.

This is an interesting finding, as little is known about the impacts of such tasks on activity in primary sensory cortices, and how neural representations in these areas may subsequently be transferred via feedforward pathways to cortical areas involved in executive functions. Although several aspects of this study are convincing and of high interest, there are some concerns regarding the analysis and interpretation of the data.

Major concerns:

1. It is unclear why the authors chose to analyze V1 as a single entity, by averaging responses and activity across the many neurons that they are able to measure, but also track in mice as they become expert at the task. Although the authors claim that timing is encoded by changes in the network dynamics, it would be interesting to know whether there still exist responses of individual cells that become tuned to specific durations (or the lack thereof, showing stochastic activity across similar trial types), whether responses could become outcome-specific with learning, the proportion of these neurons. Please provide more detailed descriptions of single cell activity pattern. For example, did each cell in V1 show reliable temporally structured activity across trials? Or did they show randomized activity across trials and their activity became meaningful only on average? Are there any cells showing ramping or phasic activity? Please add some representative plots of the activity of single cells across trials to illustrate their behavior in this task.

More generally, please show more single neuron activity examples throughout the manuscript. For instance, in Figure 3F and 3H, it is unclear what to make of Mean Network Activity and how to interpret the changes between the Naïve and Learned states. There are a wide range of scenarios that could explain these results, and the heatmaps are hard to read since the sorting is not kept across all outcomes and learning states (they are also on a different scale, some from 0 to 1.4s, some from 0 to 4s).

It is also intriguing that in Figure 6, a classifier can be used to decode stimulus type using spiking activity (6A), but that 1 individual neuron (if picked correctly) can reach the same decoding power (6H). It would mean that the variance during that 1s period can be explained by that 1 neuron, and so that the other 99 neurons are virtually silent. Is that intuition correct? Especially since the authors use a feature selection script, what is so special about the individual neurons being selected?

2. Is the neural activity in V1 encoding the timing behavior per se, or another time-dependent action? Since the animal is running on the ball, and performing go/no-go licking behavior for reward, it is possible that on hit vs CR trials, differences in neural activity could be due to differences in approach behavior (i.e. velocity on the ball), rather than reflective of timing per se. The authors should address this alternative explanation by looking at velocity on the ball 1) across trial types to test whether there is no differences in velocity despite differences in neural temporal dynamics and/or 2) within trial types to see if variation in velocity across hit trials is not explained by temporal neural dynamics.

3. It seems that some arguments were not fully supported by quantitative analysis. It would be useful if the authors could perform a few additional statistical tests on their arguments:

- One of the main results of this manuscript is that neural network activity is suppressed for ‘CR’ but not for ‘Hit’ in learned session (Figure 3H). Corresponding statistical testing of this result was given in Figure 4 by comparing the distribution of max spiking. However, the distributions of max spiking were also significantly different between outcomes in naïve session (Figure 4A, C), which contradicts Figure 3F. It would help to add an analysis directly testing the suppression vs. sustained activity at right before the choice period (i.e., 1∼1.2s).

- According to the results and abstract, Figure 3G, H suggests that the network activity was temporally coordinated with the preferred stimulus. However, there was no quantitative or statistical analysis supporting that argument. One can see an increase of ‘Hit-learned’ at the time of preferred stimulus in Figure 3H but are these bumps really strong enough (e.g., stronger than chance level)? Could the authors provide quantitative evidence on this point if this is one of the main results?

- Is the correct rejection rate actually increasing over sessions? this appears to increase by comparing naïve to learned in Fig 1 but is this due to comparing session 1 vs last day, or is there a real learning curve over days? The authors should perform a repeated measures ANOVA, or similar non-parametric test, to determine whether learning is in fact occurring over the course of training.

Minor concerns:

1. In Figure 1, the behavior profiles were given in d’ and z-scored values. In addition to these measurements, could the authors please plot them in raw values (i.e., correct percentage) as well? It would be helpful to understand the performance level.

2. Color scale bars are missing for heatmaps. Eg. Figure 3.5

3. Please provide methods for Figure 5 analysis. How can these graphs indicate the difference between trial types? Also a signifier in the plot should be used to mark different response types.

4. Please provide methods for establishing individual lick rate thresholds and their distribution.

5. In Figure 1-1A and 1-2A, were the mice in learned group and in control group the same mice? If so, then a Wilcoxon signed-rank test would be the most appropriate choice for these comparisons. (If the data satisfied the assumption for parametric test, then a paired t-test would be fine.)

6. Throughout all the figures, it would be good for the reader to know exactly what is being plotted on the Y-axis: Probability of X, Accuracy of Y, since there are many variables being analyzed (licking, activity, decoding accuracy, etc)

7. Error bars or confidence intervals are almost always missing.

8. Scale bars are missing in Fig3E and 3G.

9. The legends corresponding to figures 3-5 and 6-1 are wrong (they are switched).

Finally, kudos to the authors for a very well written discussion that offers a large range of hypotheses for potential future research.

## Author Response

We sincerely appreciate the constructive feedback from all the Reviewers regarding our submission to eNeuro. Their expertise generated very useful comments and suggestions, and by addressing their feedback the revised manuscript is much improved. It was encouraging to hear that the reviewers found this an “interesting finding” and shared our enthusiasm that this study will impact how neural representations in sensory cortical areas will influence downstream brain stream brain regions involved in higher order cognition. In the response below, we address their principal concerns, by including additional raw traces for each trial outcome (Revised Fig. 3), by expanding on our analysis techniques, by ensuring all figures are supported by statistical analyses and by including additional details in the methods to explain our analysis in Fig. 6. In the revised manuscript, we provide additional details on the analyses and statistics. The revised figures are mentioned in the rebuttal. We include Reviewer figures in the rebuttal to support our answers. Of course, we also address all of the Reviewers’ individual comments point by point. All changes to the manuscript are indicated in blue. Revised figures include ‘Revised’ in the figure name. We indicate in the manuscript that all code used for the SVM analysis will be available upon request. We hope the Reviewers will now find the manuscript suitable for publication.

Major concerns:

1. It is unclear why the authors chose to analyze V1 as a single entity, by averaging responses and activity across the many neurons that they are able to measure, but also track in mice as they become expert at the task. Although the authors claim that timing is encoded by changes in the network dynamics, it would be interesting to know whether there still exist responses of individual cells that become tuned to specific durations (or the lack thereof, showing stochastic activity across similar trial types), whether responses could become outcome-specific with learning, the proportion of these neurons. Please provide more detailed descriptions of single cell activity pattern. For example, did each cell in V1 show reliable temporally structured activity across trials? Or did they show randomized activity across trials and their activity became meaningful only on average? Are there any cells showing ramping or phasic activity? Please add some representative plots of the activity of single cells across trials to illustrate their behavior in this task.

• We want to clarify that figures where we plot average activity was done to provide a visualization of any population activity level changes that emerged. We have now included 95% confidence intervals (shaded area for each average activity plot). While figures showing cumulative distribution analyses used single cells as an individual unit, SVM and PCA analyses used the entire network. However, in Reviewer Fig. 1 we now show SVM analyses using single cells and this analyses revealed reduced accuracy compared to the analyses that utilized the entire network

• We agree that it would be interesting to characterize any duration specific cells. In agreement with theoretical models and published experimental evidence, our data supports the idea that time in the millisecond range is encoded in population activity.

We did not observe duration specific cells that reliably reproduced the 0.2 or 0.9s duration, although the distribution of all the recorded cells (Revised Fig. 3 and Fig. 4) showed differences between the stimulus condition and trial outcome.

More so, we used the SVM to compare the accuracy of single cells to that of the entire network of cells. Each individual cell’s activity is used in each time bin to discriminate Hit from CR or Hit from FA trials. Like previously, we perform 1000 iterations to produce a distribution of accuracy percentages. To compare individual cell accuracy to network-level accuracy, we use the cells with the highest accuracies for each mouse per time bin in each category of cells. For example, in the 4 cell category, we would take the most accurate 4 cells per mouse for a given time and then average all cells to produce an accuracy measure for that given time bin.

We find that individual cell activity is not sufficient to account for learning (Review Fig. 1). There is high accuracy in individual cells in naive days in Hit vs CR and Hit vs FA tests when performance is poor. Additionally, there remains high accuracy in individual cells in Hit vs FA tests in learned sessions despite incorrect responses. Comparing Hit vs CR trials between individual cell predictivity and network predictivity in learned sessions shows that network activity is an earlier and better predictor of trial outcome than are individual cells’ activities.

• We additionally failed to observe any ramping activity however there were hints of phasic activity as seen in the average activity plots in Revised Fig. 3H. We performed a Fourier analysis to determine if, after learning, the neural dynamics contained a resonant frequency. However, this analysis did not result in anything conclusive (see Reviewer Fig. 2)

• The reviewer’s intuition is correct that overall the cells’ firing was stochastic. And as shown in Reviewer Fig. 1, network activity showed higher accuracy in predicting trial outcome (likely by differentiating between stimulus types).

• When we examine changes at the level of neural dynamics, our reasoning is that single cell activity isn’t quite as simple as response fidelity/ reliability via stimulus presence or absence. However, we performed additional analysis to determine the reliability of a cell’s firing - that is, whether it has a chosen time or set of times in which it fires - we calculated Shannon Entropy values for each cell based on trial outcome and found that, with learning, there was relative uniformity across cells in stochasticity of firing times (Reviewer Fig. 3). Therefore, while our data suggest that timing is encoded in changes in neural dynamics, Reviewer Fig. 3 suggests that even at the level of individual cells there was an increase in response fidelity. In the naive session in the preferred window, all entropies were significantly different (Wilcoxon rank sum test, new alpha = .0083; Hit vs Miss: p = 1.98*10-23; Hit vs CR: p = 1.07*10-143; Hit vs FA: p = 1*10-71; Miss vs CR: p = 7.9*10-15; Miss vs FA: p = 1.13*10-20; CR vs FA: p = 4.45*10-23). In the learned session in the preferred window, hit entropies were significantly different from CR and FA entropies (Wilcoxon rank sum test, new alpha = .0167; Hit vs CR: p = 6.07*10-60; Hit vs FA: p = 6.81*10-46; CR vs FA: p = .1438). In the naive session in the nonpreferred window, CR and FA entropies were significantly different (Wilcoxon rank sum test, p = 7.49*10-94). In the learned session in the nonpreferred window, CR and FA entropies were significantly different (Wilcoxon rank sum test, p = 9.11*10-13).

More generally, please show more single neuron activity examples throughout the manuscript. For instance, in Figure 3F and 3H, it is unclear what to make of Mean Network Activity and how to interpret the changes between the Naïve and Learned states. There are a wide range of scenarios that could explain these results, and the heatmaps are hard to read since the sorting is not kept across all outcomes and learning states (they are also on a different scale, some from 0 to 1.4s, some from 0 to 4s).

We thank the reviewer for this suggestion. We do show a few traces in original Fig. 3D, however we now include additional sample traces from each of the trial outcomes (see Revised Fig. 3, included in the manuscript files)

• We now clarify the rationale for showing the heat maps on different scales. The time scale on the x axis reflects our behavior paradigm, where the trial duration for the preferred condition was 1.4s and for the nonpreferred condition it was 4.2s. Thus, stimulus offset in the preferred condition is t = 1.4 s, and stimulus offset in the nonpreferred condition is t = 4.2 s. Hit trials relate to the preferred condition and CR and FA relate to the nonpreferred conditions, so heat maps showing data from the Hit trials have an x axis of 0 to 1.4s, while the x axis for CR and FA was 0 to 4.2s.

• The criteria for sorting neural activity in the heat maps was consistent across all conditions and trial outcomes. We now clarify this in the methods. Activity of cells was sorted as a function of time. The top few rows of the heat maps plot cells that were active earlier in the trial, while the bottom rows of the heat map plot cells that were active later in the trial. This is consistent with sorting techniques used in other studies (Johnson et al., 2010; Goel and Buonomano, 2016; Bakhurin et al., 2017).

• We want to emphasize our goal with the heat maps and average activity plots in Revised Fig. 3 is to provide visualization of raw data. The heat maps show distributions of neural activity. These distributions are similar across trial outcomes in the naïve condition; however, in the learned condition different distributions emerge across the trial outcomes. This difference is statistically evaluated, across both time scales (1.4s and 4.2s), in the cumulative distributions in Fig. 4. In addition, we now include statistical analysis of mean neural activity in Revised Fig. 3, Revised Fig 3-1, and Revised Fig 3-2 in which 95% confidence intervals are plotted.

Further, extensive analyses in the subsequent figures include detailed statistical analyses and examine some of the mechanistic “scenarios” that contribute to encoding of temporal patterns.

• The method of sorting of the heatmaps is the same for the Preferred and Nonpreferred stimulus periods. It is rather that in the Nonpreferred stimulus periods there are more times in which a cell may fire, hence the different x-axes.

It is also intriguing that in Figure 6, a classifier can be used to decode stimulus type using spiking activity (6A), but that 1 individual neuron (if picked correctly) can reach the same decoding power (6H). It would mean that the variance during that 1s period can be explained by that 1 neuron, and so that the other 99 neurons are virtually silent. Is that intuition correct? Especially since the authors use a feature selection script, what is so special about the individual neurons being selected?

• We agree with the reviewer’s intuition. Fig. 6A uses all neurons collected in a given mouse to distinguish the two stimuli. Figs. 6G-H over time select a cell or group of cells to do the same. For each point in time in Figs. 6G-H, we train a classifier using either a single cell’s or group of cells’ activity to distinguish the two stimuli. This is the feature selection algorithm. So for each point in time, we can think of the neurons (features) selected as those most informative in discriminating the two stimuli. However, the feature selection does not seem to depend exclusively upon the mere presence or absence of activity, as demonstrated in Reviewer Fig. 4. We can reconstruct the activity profiles based upon which cells are selected as a function of time. We do this by taking the distribution of cells selected by the feature selection algorithm per a given time. For instance, at t = 0 s in the 1 Cell category, there are 1000 total cells selected for a given mouse as we generate 1000 classifiers, and accordingly test them, for a given mouse. We then take averages of each cell’s activity for all trials of a particular category (e.g. ‘Preferred’) and then weight that activity value by the percentage of times that cell was selected.

We then add together all the cells’ weighted activities to generate a total activity value. For instance, if the same cell was selected all 1000 times, it’s activity would get a 1 and all other cells would get a 0; thus, the entire activity of that time point would be determined by that 1 cell’s average activity. All time points are then reconstructed in this way. Reviewer Fig. 4 shows said reconstructions, one of average activity in the 1 Cell category, and 1 of average activity in the 64 cell category:

Although the activity curves in the learned session are much close together than those in the naive session, the learned session yields far more predictability. The predictability, i.e. the divergence in the activity profiles, is not simply a matter magnitude, but likely high-dimensional encoding strategies.

The methods section now includes details of the SVM analysis performed in Fig. 6.

2. Is the neural activity in V1 encoding the timing behavior per se, or another time-dependent action? Since the animal is running on the ball, and performing go/no-go licking behavior for reward, it is possible that on hit vs CR trials, differences in neural activity could be due to differences in approach behavior (i.e. velocity on the ball), rather than reflective of timing per se. The authors should address this alternative explanation by looking at velocity on the ball 1) across trial types to test whether there is no differences in velocity despite differences in neural temporal dynamics and/or 2) within trial types to see if variation in velocity across hit trials is not explained by temporal neural dynamics.

We agree with the reviewer that a general consensus in the field is that neural activity associated with sensory guided tasks can be influenced by movement and arousal states. Indeed, to circumvent this issue we removed the influence of licking by eliminating any cells throughout our analysis that were modulated by licking. We find that even after removing any lick modulated neurons, the neural dynamics reflect changes in temporal structure of the experienced stimuli (Revised Fig. 3, Fig. 4, Revised Fig. 5, Fig. 6) .

However, as suggested by the reviewer, neural dynamics and distributions could be contaminated by movement of the mouse on the ball. We do want to emphasize that before the mice begin training on the TPSD task, there is a rigorous period of pretrial to allow mice to habituate to the imaging rig, learn to lick reliably to reduce any changes in licking attributed to reduced motivation, and at the end of the pre trial phase mice are running consistently throughout the TPSD. Therefore, we did not expect to see changes in locomotion that contributed to changes in neural distribution between trial types. However, we have performed additional analyses to support our prediction- Reviewer Fig. 5 shows neural data and locomotion data. We do not see any trends for differences in locomotion that might contribute to trial outcomes.

3. It seems that some arguments were not fully supported by quantitative analysis. It would be useful if the authors could perform a few additional statistical tests on their arguments:

- One of the main results of this manuscript is that neural network activity is suppressed for ‘CR’ but not for ‘Hit’ in learned session (Figure 3H). Corresponding statistical testing of this result was given in Figure 4 by comparing the distribution of max spiking. However, the distributions of max spiking were also significantly different between outcomes in naïve session (Figure 4A, C), which contradicts Figure 3F. It would help to add an analysis directly testing the suppression vs. sustained activity at right before the choice period (i.e., 1∼1.2s).

• In Fig. 4A, the significant differences in the curves are between trials in which there is a response (Hit or FA) and when there is not (CR and Miss). In Fig. 4B, the significant differences are based upon stimulus (Hit vs CR and FA). Fig. 4C does show differences between Naive CR and FA responses; however, this is a small difference if we examine the effect sizes.

• Additionally, as in response to an earlier comment, we can reconstruct activity curves from SVM outputs in the same method to determine to what extent there is suppression. Reviewer Fig. 6 shows activity curves reconstructed using 64 cells for classification in which we classify Hit and CR responses. We use 64 cells as this captures a majority of network activity in each mouse, but still drops a considerable amount (mean = 42, std = 37). As can be seen, there is marked suppression in the CR activity (beginning at ∼.8 s) and sustained activity in the FA condition.

• Reviewer Fig. 7 shows accuracy percentages of the SVM classifying Hit and CR trials, from which the above graphs are reconstructed, shows high accuracy in the learned day and chance accuracy in the naive day, validating that learned session activity is suppressed in the CR condition.

- According to the results and abstract, Figure 3G, H suggests that the network activity was temporally coordinated with the preferred stimulus. However, there was no quantitative or statistical analysis supporting that argument. One can see an increase of ‘Hit-learned’ at the time of preferred stimulus in Figure 3H but are these bumps really strong enough (e.g., stronger than chance level)? Could the authors provide quantitative evidence on this point if this is one of the main results?

• To address this issue we performed correlations between Preferred intrastimulus on/off periods with average network activity. In the learned day, Hit trials were significantly correlated (r = .0134, p = 8.33x10^-6), CR trials were not (r = -.004, p = .1892), and FA trials were not (r = .0077, p = .0112). In the naive day, there were only significant negative correlations, i.e. no meaningful correlations: Hit (r = -.008, p = .0073); Miss (r = -.0123, p = 1.76x10^-12); CR (r = -5, p = .8); FA (r = -.021, p = 1.31x10^-12). Thus, learned day hit trials are temporally coordinated with the Preferred stimulus. (These analyses are included in the main text)

- Is the correct rejection rate actually increasing over sessions? this appears to increase by comparing naïve to learned in Fig 1 but is this due to comparing session 1 vs last day, or is there a real learning curve over days? The authors should perform a repeated measures ANOVA, or similar non-parametric test, to determine whether learning is in fact occurring over the course of training.

• We did indeed perform statistical tests here (Fig. 1D) but found no significant results. Fig. 1E represents the change in starting performance to finishing performance, as the reviewer noted. We believed that this behavioral change added clarification to the learning profiles of mice.

Minor concerns:

1. In Figure 1, the behavior profiles were given in d’ and z-scored values. In addition to these measurements, could the authors please plot them in raw values (i.e., correct percentage) as well? It would be helpful to understand the performance level.

• We elected to use d’ and z-score values in lieu of raw scores because they provide a less biased measure of performance. This is particularly important for our paradigm as the Preferred to Nonpreferred stimulus ratio is already biased. A mouse could lick 100% of the time, get all Preferred stimuli correct, all Nonpreferred stimuli incorrect, but still receive a score of 70%, which does not accurately reflect the mouse’s performance. We use d’ primarily due to Signal Detection Theory. It is used commonly in assessing performance in human and non-human animal research(Carandini and Churchland, 2013; Peron et al., 2015; Poort et al., 2015; Goel et al., 2018; Gallero-Salas et al., 2021).

2. Color scale bars are missing for heatmaps. Eg. Figure 3.5

Thank you for pointing this out. We have now included the color scale bars

3. Please provide methods for Figure 5 analysis. How can these graphs indicate the difference between trial types? Also a signifier in the plot should be used to mark different response types.

• These graphs are Principal Component Analyses (PCA) of average network activity. We average each cell’s activity over all trials and then perform a PCA using the MATLAB function pca. We then plot the trajectories of the first 3 principal components to generate these figures. These figures represent the state of the network at a given point in time. The spatial divergence between two states at a given time represents the magnitude of their commonality. They are a representation of a state-dependent network, which is, as discussed in the introduction and discussion, a prominent theory in subsecond and second timing. Previous studies have similarly characterized network activity (Levy et al., 2019; Motanis and Buonomano, 2020). We now include additional details in the methods.

• We also include arrows to indicate the trial types on the PCA plots. (Revised Fig. 5)

4. Please provide methods for establishing individual lick rate thresholds and their distribution.

• We now include additional details about how the lick threshold were calculated.

• Licking thresholds for each mouse was determined by using the average licking in the last Pretrial session minus 1 standard deviation.

5. In Figure 1-1A and 1-2A, were the mice in learned group and in control group the same mice? If so, then a Wilcoxon signed-rank test would be the most appropriate choice for these comparisons. (If the data satisfied the assumption for parametric test, then a paired t-test would be fine.)

• This is the test we performed on these data. If normal distributions, we used a t-test. If not, a Wilcoxon signed-rank test.

6. Throughout all the figures, it would be good for the reader to know exactly what is being plotted on the Y-axis: Probability of X, Accuracy of Y, since there are many variables being analyzed (licking, activity, decoding accuracy, etc)

• We tried to be very thorough with including this information in the legends. We have now made sure that indeed the legends are detailed.

7. Error bars or confidence intervals are almost always missing.

• We have now included error bars in Revised Figures 2 and 3. However as explained in an earlier comment many of the panels in Revised Figures 2 and 3 provide a visualization or conceptual impression. For example, quantification of the profiles is achieved via the SVM in Revised Fig. 2E. And quantification of differences in neural dynamics is performed in Fig. 4.

• Revised Fig 3 F&H we have changed to include s.e.m. Aside from Fig. 6 G&H we include s.e.m. in all of the SVM accuracy outputs. We elected to not include s.e.m. in those two figures for readability, however they have comparable s.e.m.’s as shown in Reviewer Fig. 8:

8. Scale bars are missing in Fig3E and 3G.

• We have addressed this in the manuscript

9. The legends corresponding to figures 3-5 and 6-1 are wrong (they are switched).

• We have addressed this in the manuscript

Finally, kudos to the authors for a very well written discussion that offers a large range of hypotheses for potential future research.

• We thank the reviewers for appreciating our efforts in making the discussion expansive and in-depth.

-

Bakhurin KI, Goudar V, Shobe JL, Claar LD, Buonomano DV, Masmanidis SC (2017) Differential Encoding of Time by Prefrontal and Striatal Network Dynamics. J Neurosci 37:854-870.

Carandini M, Churchland AK (2013) Probing perceptual decisions in rodents. Nat Neurosci 16:824-831.

Gallero-Salas Y, Han S, Sych Y, Voigt FF, Laurenczy B, Gilad A, Helmchen F (2021) Sensory and Behavioral Components of Neocortical Signal Flow in Discrimination Tasks with Short-Term Memory. Neuron 109:135-148 e136.

Goel A, Buonomano DV (2016) Temporal Interval Learning in Cortical Cultures Is Encoded in Intrinsic Network Dynamics. Neuron 91:320-327.

Goel A, Cantu DA, Guilfoyle J, Chaudhari GR, Newadkar A, Todisco B, de Alba D, Kourdougli N, Schmitt LM, Pedapati E, Erickson CA, Portera-Cailliau C (2018) Impaired perceptual learning in a mouse model of Fragile X syndrome is mediated by parvalbumin neuron dysfunction and is reversible. Nat Neurosci 21:1404-1411.

Johnson HA, Goel A, Buonomano DV (2010) Neural dynamics of in vitro cortical networks reflects experienced temporal patterns. Nat Neurosci 13:917-919.

Levy DR, Tamir T, Kaufman M, Parabucki A, Weissbrod A, Schneidman E, Yizhar O (2019) Dynamics of social representation in the mouse prefrontal cortex. Nat Neurosci 22:2013-2022.

Motanis H, Buonomano D (2020) Decreased reproducibility and abnormal experience-dependent plasticity of network dynamics in Fragile X circuits. Sci Rep 10:14535.

Peron SP, Freeman J, Iyer V, Guo C, Svoboda K (2015) A Cellular Resolution Map of Barrel Cortex Activity during Tactile Behavior. Neuron 86:783-799.

Poort J, Khan AG, Pachitariu M, Nemri A, Orsolic I, Krupic J, Bauza M, Sahani M, Keller GB, Mrsic-Flogel TD, Hofer SB (2015) Learning Enhances Sensory and Multiple Non-sensory Representations in Primary Visual Cortex. Neuron 86:1478-1490.
